# Global subnational estimates of migration of scientists reveal large disparities in internal and international flows

**DOI:** 10.1073/pnas.2424521122

**Published:** 2025-04-11

**Authors:** Aliakbar Akbaritabar, Maciej J. Dańko, Xinyi Zhao, Emilio Zagheni

**Affiliations:** ^a^Department of Digital and Computational Demography, Max Planck Institute for Demographic Research, Rostock 18057, Germany; ^b^Department of Sociology, Leverhulme Centre for Demographic Science, University of Oxford, Oxford, United Kingdom OX1 1JD; ^c^Adaptive Rationality Center, Max Planck Institute for Human Development, Berlin 14195, Germany

**Keywords:** subnational scientific mobility, internal migration, international migration, bibliometric data

## Abstract

Researchers are key contributors to innovation. Their migration results in talent circulation and recombination of ideas. Due to data shortage, little is known about subnational mobility of scientists and the interrelationships between their internal and international migration patterns. We used data on 30+ million Scopus publications of 19+ million authors to infer migration from changes in affiliations. Our publicly shared database of global subnational estimates of migration reveals relevant disparities in the attractiveness of subnational regions. While, at the country level, some countries have acted as a global hub that attracts scholars from throughout the world, at the subnational level, some of their regions have negative net migration rates, with implications for scientific output and regional development, as well as the perpetuation of migration corridors. On average, subnational inequalities in attracting and sending scholars have increased for international but decreased for internal migration. In most countries, there is no single trend such that all subnational regions have been sending or receiving more scholars. Instead, a mix of patterns has been simultaneously at work, on the backdrop of globalization of migration, which is an asymmetric process where specific regions and subpopulations have higher access to international migration. For most subnational regions, when they are attractive for international migrants, they are also attractive for internal migrants, which is not always the case for emigration. Our results have implications for the global circulation of academic talent by adding the dimension of internal migration to “brain drain” and “brain circulation” in international migration.

## Significance

Migration of scientists contributes to circulation of ideas and innovation. While this is the case for both migration within a country (internal) and across countries (international), most research has focused only on international migration, due to lack of data. We generated a global database of migration of researchers that includes internal mobility and enables us to study the interrelationships between internal and international migration, and how they affect each other. We document important inequalities across subnational regions in terms of both international and internal migration flows, and show how the two components of migration jointly determine the broader patterns of attractiveness of localized regions, within the context of the globalization of migration.

International mobility is known to favor career advancement and professional recognition of academic scientists (1). Among others, it facilitates the recombination of ideas (2, 3) and the expansion of networks of collaborators, which, in turn, lead to better science and higher visibility of scholars (4). The benefits of geographic mobility are not felt only by individual scientists. Migrant scholars tend to be a highly selected group of researchers who are particularly productive and creative to start with (5). They can promote knowledge circulation and act as an engine of growth, especially in the destination countries (2, 3). As a result, they are often the target of national policies (6, 7) aimed at attracting “the best and the brightest” (8).

The research on the determinants and consequences of migration of scholars has focused on international relocations (9, 10). However, internal migration is generally expected to be higher than international migration. It could have a bigger impact on the dynamics of populations of scholars across subnational regions (11), with consequences in terms of the vibrancy of regions and their potential for serving as hubs of discovery. Despite the importance of internal migration for the scientific vitality of regions, our knowledge of patterns of migration of scholars at the subnational level is extremely limited, and we have virtually no empirical evidence on how internal migration is interrelated with broader patterns of international migration. While we expect that the systems of internal and international migration are interconnected (12–15), understanding their dynamics has been hindered by lack of appropriate data across different groups of migrants (16–20).

In the context of migration of actively publishing researchers, bibliometric data offer previously unavailable opportunities (9, 10, 21) for further investigation of the prevalence of internal migration, and for jointly assessing the dynamics of internal and international migration processes. These joint dynamics typically cannot be assessed for other groups of migrants, including other types of high-skilled migrants, for whom we do not have detailed and longitudinal digitized information on mobility events and bilateral flows between subnational regions (21).

In this article, we propose an integrated framework that uses large-scale bibliometric data to address the gap in understanding internal and international migration systems simultaneously. Specifically, i) we quantify the extent to which academic talent circulation has happened “within” the national borders versus “between” countries; ii) which subnational regions are particularly attractive for scholars who move internally or internationally, and which ones have been losing scientists; and iii) how the dynamics of internal and international migration systems are interrelated.

## Results

### Global Subnational Estimates of Migration of Scholars Reveal Patterns of Spatial Heterogeneity.

**Fig. 1.**
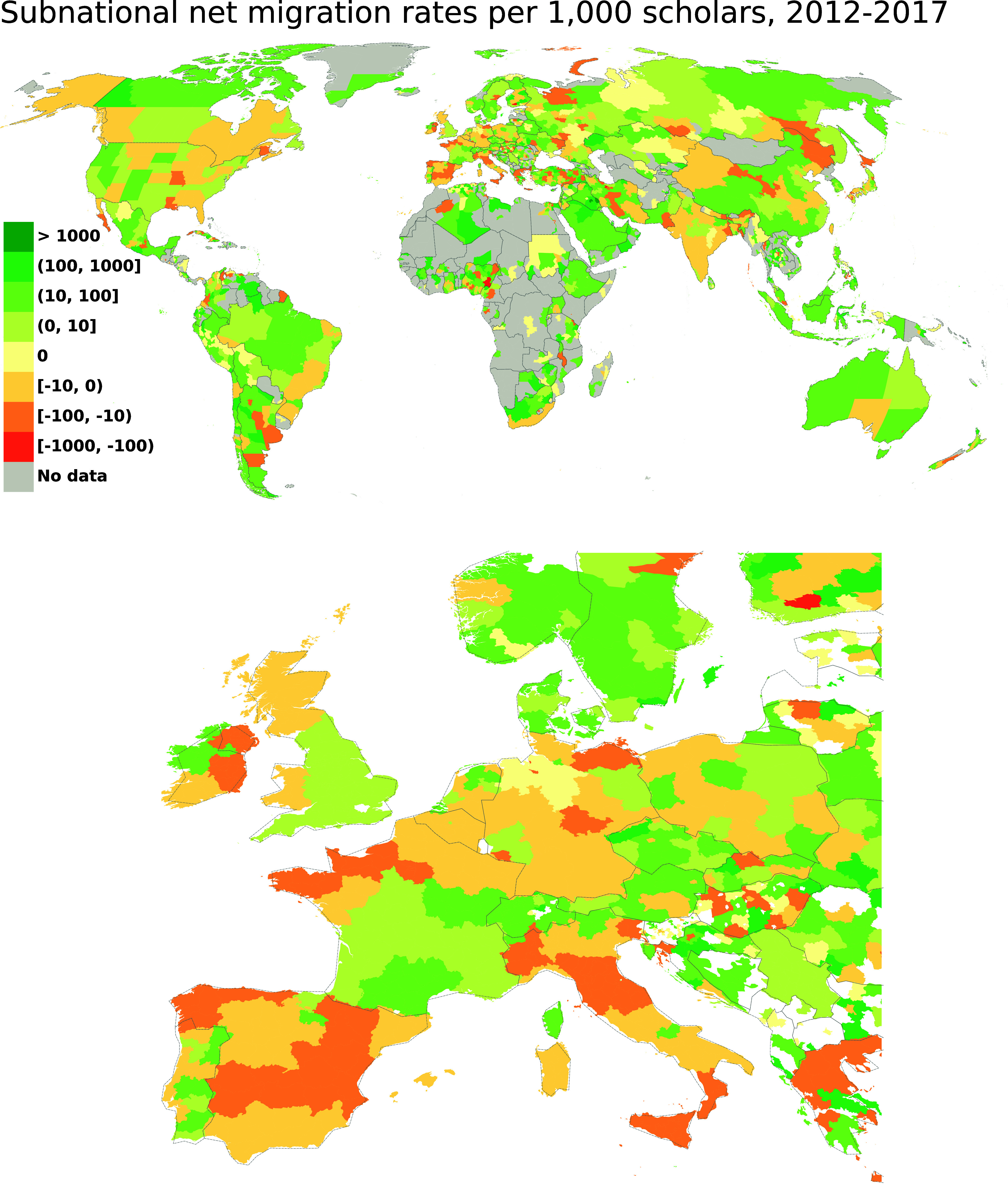
Subnational net migration rates (NMRs) per 1,000 scholars (using GeoNames admin-1), worldwide (*Top*) and for Europe (*Bottom*). The NMRs shown in the maps include both internal and international moves. Colors indicate the sign and value of the net migration rates (e.g., negative, red colors; positive, green colors). The rates are expressed per year, for the period 2012–2017, as an illustrative example of the 20 y analyzed (*SI Appendix*, Fig. S4 for the 2000–2005 and 2006–2011 periods and 1998–2017 in replication materials). Color scales are kept the same in the two maps to allow for comparison. Numbers printed on the legend are the combined net rate of scholars sent or received to/from internal and international origins/destinations, showing which subnational regions are sending more scholars than they receive or the reverse. See *SI Appendix*, Fig. S5 for separated maps for internal and international NMRs, and *SI Appendix*, Fig. S8 for disaggregation by fields of science.

We leveraged large-scale bibliometric data comprising more than 30 million publications indexed in Scopus, written by more than 19 million authors from 1996 to 2020, to estimate the migration of actively publishing researchers at the subnational level. We used disambiguated and geocoded affiliation addresses to infer migration events based on changes in institutional affiliations for unique authors over time (more details about disambiguation and treatment of authors with multiple affiliations are in *Materials and Methods* and *SI Appendix*). With this information, we produced a global database of migration of scholars covering both internal and international migration at the GeoNames Admin-1 level, over more than two decades. The aggregate-level data, openly available through the companion data repository to this article, enabled us to advance our understanding of population dynamics of scholars, at both the national and subnational levels, and could be further leveraged by the scientific community. We produced a number of indicators of migration, capturing 1) within-country (internal) migration, 2) movements to and from specific regions and foreign countries (international migration), and 3) both internal and international dimensions simultaneously. Fig. 1 shows an illustrative example of estimates produced as part of the global database, which adds subnational granularity to a previously developed database at the country level (21). More specifically, the figure shows the average net migration rates (NMR) for the period from 2012 to 2017 (See the figure for 2000–2005 and 2006–2011 in *SI Appendix* and 1998–2017 in replication materials). The rates are computed as the difference between the sum of scholars entering a specific region, and exiting the region, during the six years considered (from/to any other region, regardless of whether they are in the same country or in other countries), divided by the sum of the population of scholars in the region over the respective period of time. Thus, positive values for the NMR (marked in green shades) indicate that more scholars entered the region than left it during the period. Negative values (marked in red shades) indicate that more scholars left the region than entered it during the period. The maps in Fig. 1 provide evidence of substantial spatial heterogeneity in the attractiveness of subnational regions for scholars. For example, while the United States as a whole has had consistently positive net migration rates and has acted as a global hub that attracts scholars from all over the world (9, 10, 21), some of its regions have shown negative net migration rates, meaning that migration serves as a mechanism to reduce the number of scholars in the region. When we consider subnational regions of the United States, we observe that, across the board, they receive more scholars from abroad than they send. However, when it comes to internal movements within the United States, some of the differences are stark as regions that have positive net inflows from abroad lose a large fraction of their population of scholars to other regions in the United States, leading to overall negative migration rates (*SI Appendix*, Fig. S5). Internally, in the United States, scholars are, on average, moving from East to West, also reflecting broader demographic trends in the country where the mean center of the general population (using the centroid definition) has been progressively drifting westward. In Europe, Nordic countries tend to have positive net inflows of scholars, whereas Southern Europe, on average, has been seeing more scholars leave than move there. However, the landscape is quite heterogeneous at the subnational level. For example, while Italy as a whole has had negative net migration rates, some regions, like the autonomous “Trentino-Alto Adige,” bordering Austria, have seen substantial investments in science that in turn translated into positive inflows of scholars, internally and internationally. In France, we observe a North–South divide, with regions in the North that, on average, lose scholars, and regions in the South that gain scholars. This pattern is driven mostly by the internal migration of scholars (*SI Appendix*, Fig. S5 and a typology of regions in *SI Appendix*, Table S8). More broadly, the new estimates provide a deeper understanding of phenomena often labeled as “brain drain” or “brain circulation.” For example, India, at the country level, has a negative NMR, indicating that it sends more scholars abroad than it receives (21). However, when zooming in on subnational regions, a more complex picture emerges. We observe that some regions in India, such as central and northern ones have a positive NMR, indicating that some specific regions have served as dynamic attractors of scholars. A similar nuanced image of circulation of scholars is visible in other major players in the global science system, such as Brazil and China. These observations point to the need for a statistical investigation of the relationships between internal and international migration, which is discussed in the next subsections. Further details and disaggregations, based on fields of science, are presented in *SI Appendix*.

### On Average, Subnational Inequalities in Attracting and Sending Scholars Have Increased for International Migration, But Decreased for Internal Migration.

**Fig. 2.**
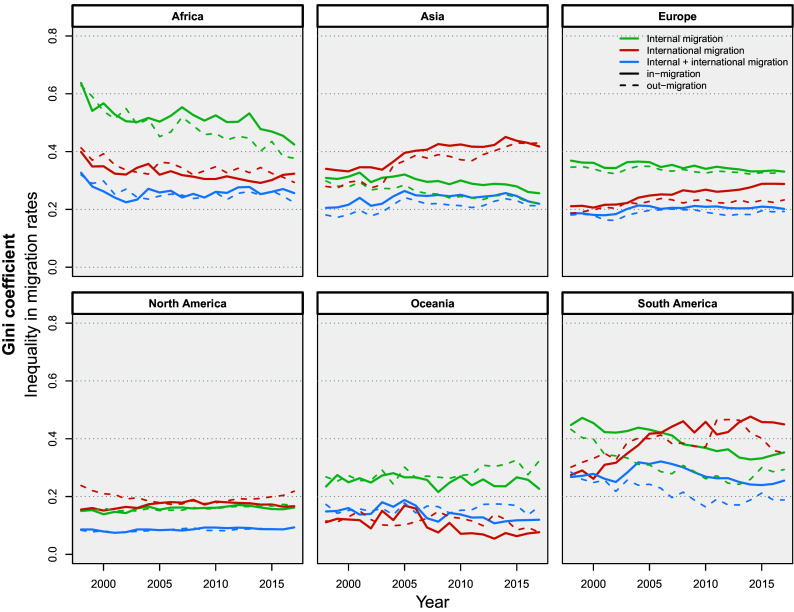
Year-specific, region-based weighted relative Gini coefficients across continents and six types of migration. Population size is used as a weight to mitigate bias arising from regions with a small number of scholars and, consequently, considerable sampling variation in migration rates, and to allow for comparison between different migration types and continents.

In the previous subsection, we provided a snapshot of spatial heterogeneity at a single point in time. Here, we examine trends in the distribution and concentration of migration flows. We calculated the relative Gini coefficient over time for in-migration and out-migration rates, both at the internal and international levels, to determine whether subnational regions are becoming more similar or more different in terms of migration scales. Fig. 2 shows the population-weighted inequality in migration rates among regions of each continent. To simplify the comparison, we presented the Gini coefficients over time at the continental level for six types of migration: internal in-migration, internal out-migration, international in-migration, international out-migration, and the combined totals for both in- and out-migration.

For subnational regions in Asia and South America, and, to a lesser extent, in Europe, we see a pattern of increasing inequality in international migration and decreasing inequality in internal migration. This means that a relatively small group of subnational regions have become more prominent in attracting and sending scholars internationally, in their respective continents, while other regions have become less dynamic. Conversely, when it comes to internal migration, subnational regions have become more similar to each other over time. For the African subnational regions for which we have enough data to compute migration rates, we observe a reduction in inequality across all types of migration, and more so in internal migration, which goes down from 0.6 (highest among all continents) to 0.4. However, given that we do not have enough data for a substantial fraction of subnational regions in Africa (as shown in Fig. 1), we cannot provide a definite picture for the continent. North America and Oceania show, in general, lower inequality in their regions (below 0.3), that stays rather steady across all types of migration over time. This means that there are relatively stable patterns of flows of incoming or outgoing migrant scholars in these continents that have not changed much during the 20 y of observation, and a fairly consistent and continuous academic talent circulation (see a typology of subnational regions in *SI Appendix*, Table S8). On the contrary, Asia and South America present a change in their Gini coefficient over time indicating that subnational regions in these continents have gone through an era of transformation in scholarly migration. This could indicate that only some regions have changed and become popular sending or receiving regions over time, leading to increased inequality between all regions in the continent. This is in line with the literature that shows that globalization of migration affects different subpopulations and geographical regions differently (22). Some subpopulations, such as the highly skilled, have better access to international migration. However, scholarly migration, similarly to the case of the general population (22), is concentrated in a shrinking set of destination countries. While the origin countries might have become more diversified, still the concentration of knowledge centers and economic activities in large metropolitan areas (23, 24) result in the subnational regions hosting the largest universities to be receiving a disproportionately larger inflow of scholars, both from internal and international origins.

### The Interrelationship Between Internal and International Migration Worldwide.

**Fig. 3.**
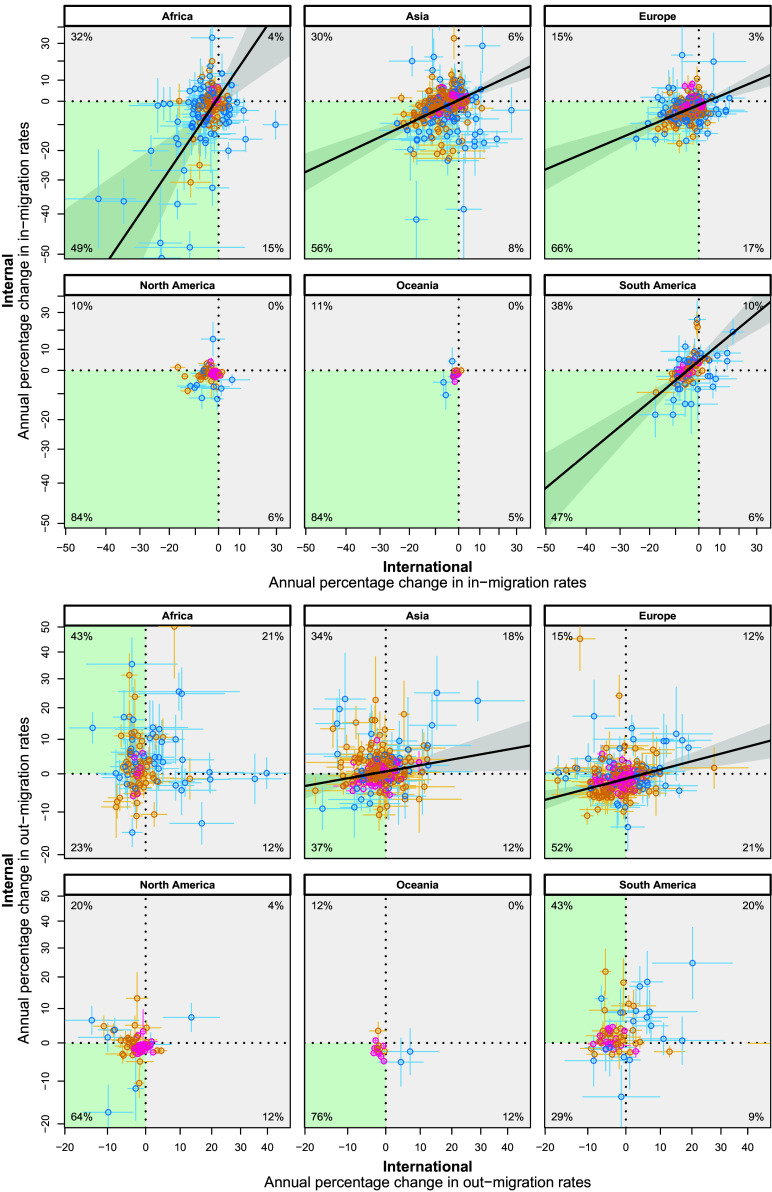
The data points, shown as circles, represent migration rate trends over time for in-migration (*Top*) and out-migration (*Bottom*) in each region. The X- and Y-axes correspond to the slopes of international and internal migration trends, respectively, derived from region-specific time trends using independent quasi-Poisson regressions. Regions are categorized by scholar population size: blue for 0 to 100 scholars, orange for 100 to 1,000 scholars, and magenta for more than 1,000 scholars. Semitransparent horizontal and vertical lines indicate the SEs for each estimate, reflecting uncertainty around the slopes. The percentages in each corner represent the fraction of regions per quadrant. The green-shaded area highlights the quadrant with the highest fraction of regions. The solid black lines in the plot represent fitted Bayesian linear models that account for SEs in both the dependent and independent variables. Shaded gray areas around the lines indicate the 95% credibility intervals. Only fits with statistically significant slopes are shown (where 95% credibility intervals of estimated slopes exclude zero). The axis values are transformed using T(x)=100×(exp(x)−1), where *x* is the original label value (the estimated slope of migration trend on the linear predictor scale), and T(x) represents the proportional change per year for easier interpretation (e.g., T(x)=20 indicates a 20% increase in the migration rate per year, T(x)=−20 indicates a 20% decrease, and T(x)=0 indicates no change); for further context, see *SI Appendix*, Fig. S12, where regions are grouped by World Bank income levels instead of continents.

To assess the relationship between internal and international migration, we computed, for each subnational region, the average trends in migration rates (out-migration and in-migration, respectively), to/from other subnational regions within the same country (internal migration) or to/from subnational regions in other countries (international migration). Positive trends indicate that the specific rates have been increasing over time. Operationally, we assessed these average trends over time by computing the slopes of quasi-Poisson regressions, estimating the temporal trends from 1998 to 2017 for in- and out-migration rates. We included all global subnational regions with more than 25 scholars in at least one observation year, and a minimum of 10 observation years with at least 5 nonzero migration counts. Fig. 3 shows patterns of interrelationships for trends in internal and international migration. The *Top* 6 panels in Fig. 3 refer to in-migration rates. The quadrant with the largest number of regions is highlighted with a green shade (for each quadrant, the percentages in the corner represent the fraction of regions in the quadrant). Across all continents, the *Bottom*-*Left* quadrant contains the highest percentage of subnational regions. It means that both internal and international in-migration, on average, have been decreasing over time for the largest fraction of regions. When we consider the fitted solid lines, we observe that, in all continents except for North America and Oceania, there is a statistically significant and positive relationship between changes in internal and international in-migration rates. This means that, on average, if a region is attractive in terms of receiving scholars, it receives scholars from both internal and international origins. The fitted trend line is shown only for statistically significant results (slope coefficient significantly different from zero), which excludes North America and Oceania. The 6 panels at the *Bottom* of Fig. 3 refer to out-migration. They show that Africa and South America have the highest percentage of subnational regions located in the *Upper*-*Left* quadrants on the Y-axis (43% for both continents). This indicates an increase in internal out-migration over time in contrast to a decrease in international out-migration. Similarly to in-migration, in Asia, Europe, North America, and Oceania, the largest proportion of regions is in the *Bottom*-*Left* quadrant (in green shade). It means that, in most regions, both types of out-migration decrease over time (*SI Appendix*, Fig. S12 for a comparison of subnational regions based on the World Bank’s income groups instead of continents). The relationship between changes in internal and international out-migration is positive and significant only in Europe and Asia. This indicates that, on a global scale, it is not always the case that those regions that experience an increase in international out-migration also experience an increase in internal out-migration.

**Fig. 4.**
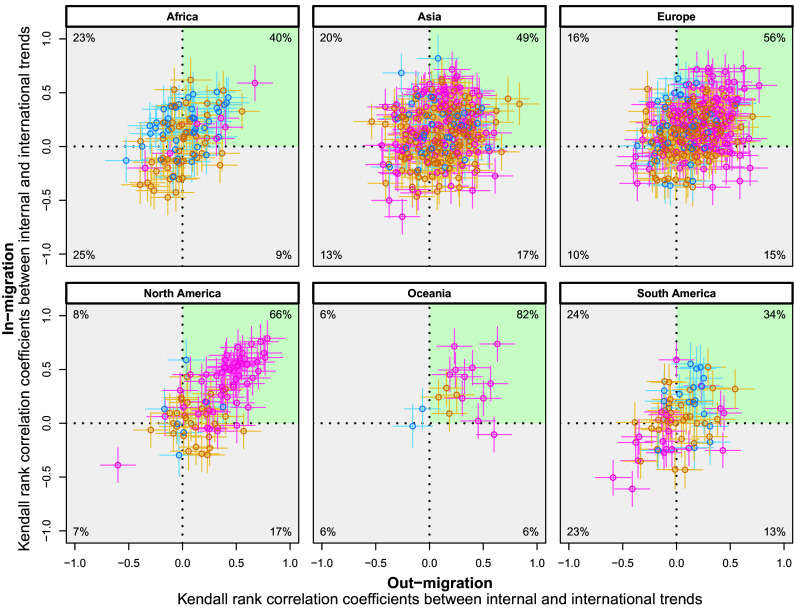
Relationships between in-migration and out-migration: each data point shows the Kendall rank correlation coefficients between internal and international (1998–2017) for in-migration (Y-axis) and out-migration (X-axis). Circles represent subnational regions where Blue shows subnational regions with 0 to 100 population of scholars, Orange 100 to 1,000, and Magenta above 1,000. The values in each corner represent the percentage of regions per quadrant. The green-shaded areas highlight the quadrants with the highest fraction of regions. As some coefficients are 0, they may not sum to 100%. The *Upper*-*Right* quadrant includes the largest percentage of subnational regions in each continent. This indicates that the correlation between internal and international migration is positive for both in-migration and out-migration rates. The *Bottom*-*Left* quadrant indicates that the correlation is negative for both in-migration and out-migration. Similar logic applies for the remaining quadrants, however, the positive correlation between internal and international is recorded only for one data type. The positive correlation between internal and international means that for both data types, there are either positive or negative time trends. The negative correlation means that for both data types, we see opposite directions in internal and international migration. See *SI Appendix*, Fig. S13 showing groups of regions based on the World Bank income levels instead of continents.

Fig. 4 shows relationships between in-migration and out-migration where each data point shows the correlation, at the subnational regional level over time, between internal and international trends. Across all continents, the largest percentage of subnational regions is in the *Upper Right* quadrant, in green shade, meaning that, for most regions, when the correlation between internal and international trends is positive for out-migration rates, it is also positive for in-migration rates. A positive correlation means that either both internal and international trends are increasing over time or both trends are decreasing in sync. In North America, the strongest correlation is observed for regions with relatively large numbers of scholars, indicated by magenta circles, which are located in the *Top Right* corner. However, there is also heterogeneity across continents, with the largest regions in some continents displaying low levels of correlation between internal and international trends.

## Discussion

In this study, we have produced a global database of scholars’ migration at both internal and international levels. This database, which builds upon prior work on international scholarly migration (21), enabled us to address a documented gap in the literature in the context of understanding the interrelationships between internal and international migration systems (14, 15) within a unified framework (12, 13, 20). On a global scale, we developed indexes of internal and international migration of scholars (11, 25) and showed that there is more nuance to the generally depicted picture of brain drain and brain grain. In most countries, there is heterogeneity in scholarly migration rates across subnational regions: we do not observe a uniform picture where all subnational regions of a country are sending or receiving scholars. Rather it is a mix of these trends that are simultaneously at work. This is in line with the literature (22) indicating that globalization of migration is an asymmetric process where specific regions and subpopulations have higher access to international migration.

We observed that some continents, such as Africa and South America, have experienced a transformation in scholarly migration trends over time, where inequality of migration rates have changed, i.e., decreased and increased, respectively. This entails that some regions have become attractive or unpopular over time, leading to changed inequality between regions. For continents with larger science systems, in terms of the number of researchers, such as North America and Oceania, we observed lower levels of inequality, which is stable over time, indicating consistent trends in scholarly migration.

A consistent pattern is observed in in-migration rates for all continents and most subnational regions worldwide. The majority of regions experience both decreasing internal and international migration rates. However, some regions show an increase in both internal and international migration rates over time, notably in South America and Asia. These regions might be attracting scholars to move in or return due to enhanced educational opportunities or favorable research environments. Alternatively, these regions could be hosting displaced migrant scholars who are (in)voluntarily choosing to move there due to unpleasant conditions such as war and conflict (26). Regions with a large number of scholars exhibited relatively smaller changes in migration rates over time and were located in the central part of the figures. In contrast, the extreme changes in migration rates of scholars were specific to relatively smaller regions. Larger regions hosting a significant number of scholars are centers of economic and knowledge activities (23, 24) and have more resources, resulting in smaller changes in migration rates over time and have a continuous flow of incoming and outgoing migration. Conversely, smaller regions might undergo more pronounced shifts, potentially due to changing local conditions. This pattern is not the case for the Kendall rank correlation measure comparing the interrelation in internal and international migration, except in North America and Oceania. In North America, the strongest correlation between internal and international migration trends is observed for the largest regions. Their in-migration rates decreased for both internal and international migration of scholars. These results may be attributed to common factors influencing both types of migration, such as research opportunities, academic programs, or economic conditions.

While similar patterns to in-migration can be found for out-migration rates in Europe, North America, Oceania, and Asia, where most subnational regions were located in the *Bottom Left* quadrant of the figure (Fig. 3, *Bottom*), indicating decreasing slope of trends over time, Africa and South America exhibit an increase in academics leaving. In these continents, most regions send scholars to internal destinations, and this trend exhibits an increasing slope over time. Additionally, most of these regions also experience decreasing international migration rates. Similar to in-migration, the larger slopes are more prominent among smaller regions with smaller populations. The observed trends suggest a significant academic movement toward internal destinations, possibly indicating the attractiveness of certain regions for scholars or a potential inequality in resource allocation leading to a concentration of resources in capitals or specific metropolitan regions (23, 24). While we present nuanced differences in trends based on the field of science (*SI Appendix*), further research on academic age and status, socioeconomic factors, and regional characteristics is needed to better understand these results. Our database enables such investigations.

We found a group of countries where internal scholarly migration was more prevalent (*SI Appendix*, Fig. S5 with aggregate net migration rates). These countries with larger science systems most likely can hire their graduates and promote them to permanent positions (27, 28). Internal scholarly migration is more prevalent and has a higher impact on these science systems. Our more detailed investigation of internal and international scholarly migration in subnational regions of these countries showed a highly dynamic trend. Some of the regions were mainly sending regions whose talent is hired elsewhere inside the country or in international destinations (see a typology of subnational regions in *SI Appendix*, Table S8).

The dynamics of talent circulation between subnational regions of the countries were insightful. While specific countries such as the United States act as an international hub of academic talent at the country level, at the subnational level, some states (e.g., mid-eastern ones) were losing scholars to other states (western ones). This implies that, while mobility enhances interregional and international collaboration, benefiting the entire scientific community, concentration of scientific talent in selected subnational regions could foster the emergence of innovation hubs. These regions would then benefit from a cumulative advantage as a critical mass of scientists attracts more talent, funding, and collaborators. Conversely, other regions may experience less potential for innovation-led growth. In addition to having an impact on scientific output and on regional economies, the emergence of disparities in international and internal migration of scholars may also have implications for patterns of migration themselves. For example, international migration to regions that are less attractive within a given country may, in some instances, represent an intermediate step toward internal migration later on to more established hubs within the country. Alternatively, positive international net migration to areas that are attractive to internationally mobile scientists, but that are not as attractive to local scientists, may serve the role of compensating for negative internal net migration rates for specific regions.

Our investigation of scholarly migration, and integrated examination of both internal and international aspects, has unveiled crucial findings regarding trends worldwide. By utilizing innovative data sources and methodologies, and by preparing a comprehensive global database, we were able to identify destinations of scholarly migration, differentiate between sending and receiving regions, and explore potential underlying factors influencing migration trends. This offers unprecedented opportunities to study internal and international systems of scholarly migration (12, 13) in an integrated framework. Further investigation of potential underlying factors [e.g., economic development (9)] driving the observed trends would be very valuable. This type of investigation is influential in the area of high-skilled migration and global talent circulation (1, 4, 29, 30).

Our study and database have certain limitations that we would like to acknowledge. Academic entities’ names (e.g., author and organization names) need disambiguation and cleaning (24, 31–33). We have used the most reliable available database (34) and have made important progress in that direction (21, 24), but there is still space for improvement in future research. Repurposing bibliometric data for migration research (21, 35, 36), and assigning the country of affiliation in the first publication as the country of origin for academic mobility could introduce error since that could be the country of graduation. Even though that country provides resources to scholars to start their publishing career, it is different from their country of birth (9). With the available data, we can infer changes of residence, but we do not have information about nationality or country of birth. Further, there is an increasing trend in authors with multiple affiliations. While our empirical investigation (*SI Appendix*) showed that our approach minimizes the potential impact of such cases, future research could use information about multiple affiliations to further model the complexity of migration and short-term mobility dynamics. Finally, these data are limited to only those researchers who have actively published in scholarly journals indexed by bibliometric databases, which, while covering other (mostly Western) languages (34), are dominated by English-speaking publications.

Despite these limitations, we curated a global database of researchers’ migration and answered some previously nonaddressable questions which were due to a lack of data on internal and international migration (12, 20, 21, 37). Our database enables addressing such questions for a subset of highly skilled migrants, i.e. scholars. Our results have implications for theories of migration, by showing that different subnational regions in a country, with a specific development history, could exhibit specific migration trajectories. We observe indications that globalization of migration is an asymmetric process affecting different regions and subpopulations differently (22). This is in line with the literature emphasizing that migration policies affect different subpopulations differently (38) and highly skilled migrants are attracted to destinations with more visa agreements, with appreciation of qualifications, and with point-based and qualification-oriented policies (39). Since the links between migration of scholars, knowledge diffusion, and collaboration are empirically and theoretically understudied (40), our analyses highlight that such links need to be studied with respect to both the internal and international dimensions.

## Materials and Methods

We use publications data from Elsevier’s 2020 snapshot of Scopus that is provided to us by the German Competence Network for Bibliometrics (41) through the Max Planck Digital Library. We limit the data to only “article” and “review” publications to have the highest quality of metadata (24, 41). After disambiguation and geocoding to the subnational level, using our previously developed methodology (24) that relies on a local installation of the Research Organization Registry Application Programming Interface, we excluded publications without usable addresses. Our dataset includes 30,757,444 publications from 1996 to 2020 by a total of 19,050,557 Scopus-published and disambiguated authors. Note that, in our statistical models, we include the data from 1998 to 2017 to reduce left- and right-censoring/truncation effects in the data (21) (more details in *SI Appendix*).

We identified subnational scientific mobility using a measure that relies on detecting changes in modal regions of affiliation over time. This approach is stable and less prone to noise and fluctuations (9–11, 21). This means that we considered all affiliation countries of a Scopus author ID in a single year. It also considers authors with multiple affiliations (more details in *SI Appendix*). If there is more than one country in a year, we take the *mode* (the most frequent affiliation) of all countries, and in the case of multiple modes, we choose the one that was present in the closest previous years. When all mode countries are unique and new, we choose one randomly (9, 21). A migration event is recorded when the mode country of residence in year *t* changes for the year of the next observation. The same happens for the subnational regions where the residence is the mode region in a given year. Additionally, since our analysis is at the subnational level, for international migration, the pair of regions considered are in two different countries, while for internal migration, this pair includes two distinct regions inside the same country. Furthermore, we assume a *two-year* preparation time for all publications to cover disciplinary differences in publication delay (42). If there are gaps in publication years (e.g., authors are not publishing continuously), we backward fill each publication year for two years and assume the author’s residence to have been changed two years earlier (21). If there is enough evidence (i.e., continuous publication activity), we consider the year a modal affiliation changes as the migration year. Please note that, in a strand of the science of science literature, migration is considered a longer-term mobility event (43), whereas for shorter-term moves, labels such as *workplace mobility* have been used (44). Here, based on the mode-based definition, identification of migration events needs observations over the course of a minimum of two years. Hence, we are interested in longer-term moves rather than temporary stays, which might not result in a change of academic affiliation. In the manuscript, we use the words *mobility* and *migration* interchangeably. An underlying assumption of the approach is that the center of one’s professional life (the geographic location of their institutional affiliation) should be aligned with one’s center of personal life (their main residence). Assessing mobility for people with more than one residence, or with extremely long commutes, is an ongoing area of investigation in spatial analysis beyond scholarly migration. As this issue could affect estimates for small areas, like cities, we kept our analyses at a geographic level (e.g., countries, and macrosubnational regions) for which the potential impact of this issue on estimates should not be substantial.

To aggregate the count of migration events to the region, country, and continent levels, we calculated different measures. We calculated the NMR, as in Eq. **1**, that accounts for the incoming and outgoing population of scholars over the total number of scholars in a region or country in a given year:

[1] $$NMR_{i,t,k}=1,000\times \frac{I_{i,t,k}-E_{i,t,k}}{N_{i,t}}$$

where *i* is the subnational region. *t* is the year. Subscript *k* shows the type of data, i.e., internal or international. $I_{i,t}$ is the inflow of scholars entering a region and $E_{i,t}$ is the outflow of scholars exiting that region. $N_{i,t}$ is the total number of scholars in the region in a given year. We present results based on NMR of internal and international scholarly mobility to highlight the interdependence between these two systems of migration (12, 13) while in *SI Appendix*, we present the separated figures and additional ones disaggregated by field of science.

### Statistical Methods.

To measure inequality in migration rates across subnational regions, we calculated the weighted Gini coefficient for each type of migration within each continent. The Gini coefficient was computed annually, using the population size of each region as weights (45).

Slope coefficients shown in Fig. 3 were estimated separately for each region to capture general time trends. We modeled migration counts as the dependent variable using a quasi-Poisson regression with a log link function, including an offset term for the log number of scholars in a given region and year. The quasi-Poisson model was chosen to account for overdispersion, which is common in count data and can lead to underestimated SEs in standard Poisson regression.

The estimated slopes and their SEs were then used as inputs for a Bayesian linear regression model, which explicitly accounts for measurement uncertainty in both the dependent and independent variables by incorporating their SEs. The posterior mean estimates from the Bayesian model are shown as solid black lines, with shaded areas representing the corresponding 95% credible intervals (shown only for statistically significant results where credible interval excludes zero). The model was fitted using the brms R package (46, 47).

The Kendall rank coefficient (48) was used to measure the ordinal association between two variables and is based on ranking the elements of the sample. In *SI Appendix*, Figs. S10 and S11, we calculated Kendall rank coefficients independently for each regional trend. In Fig. 4, the Kendall rank coefficients were also calculated for each region separately, and we used them to measure the association between internal and international migration rates. Migration rates were calculated as counts of migration events divided by the number of scholars.

For additional details on statistical methods, please refer to *SI Appendix*.

## Supplementary Material

Appendix 01 (PDF)

## Data Availability

All scripts, data, and materials to replicate our analysis and figures are deposited in the permanent repository available at this link https://doi.org/10.5281/zenodo.15047101 (49).
