# Supplementary material for: Global subnational estimates of migration of scientists reveal large disparities in internal and international flows
Source: Proc Natl Acad Sci U S A. 2025 Apr 11;122(15):e2424521122. doi: 10.1073/pnas.2424521122 (PMC12012457; doi:10.1073/pnas.2424521122)
Supplement: Supplementary file 1 — Appendix 01 (PDF) [file pnas.2424521122.sapp.pdf]

Supporting Information for

Global subnational estimates of migration of scientists reveal large disparities in internal and international flows

Aliakbar Akbaritabar, Maciej J. Dańko, Xinyi Zhao, Emilio Zagheni

Corresponding Author Aliakbar Akbaritabar.
E-mail: akbaritabar@demogr.mpg.de

This PDF file includes:

Supporting text
Figs. S1 to S13
Tables S1 to S8
SI References

Supporting Information Text

Supplementary methods and results. Here we present further information on our data, pre-processing steps needed, and additional results on specific country cases to complement the results included in the main text.

More on data and pre-processing steps. We used publications data from Elsevier's 2020 snapshot of Scopus that is provided to us by the German Competence Network for Bibliometrics (1). It includes 30,757,444 "article" and "review" publications by 19,050,557 researchers from 1996 to 2020. We limit the publication types to only these two in order to have the highest possible accuracy of metadata based on the previous evaluations by (1) and ourselves (2–4). Re-purposing publication data for our goals requires extensive data processing and cleaning. Further, bibliometric data needs fine-grained cleaning, encoding countries and geographical regions of affiliation. Academic organization and author names need to be disambiguated since, for author names, homonyms and name changes occur, and for organizations, spelling errors or use of different order of name parts happen (5). For author names, we use Scopus's author IDs, with 98.3% precision (no publication by others is included in X's publication list) and 90.6% recall (all publications by X are included) as reported by (6). For the academic affiliations and organization names, we use our previously developed methodology (7), which uses the Research Organization Registry (ROR) application programming interface (API). By sending affiliation strings from Scopus to ROR API, we can identify similar affiliations with spelling and name order differences, and group them under unique addresses. In addition, after processing bibliometric data, we complement them with GeoNames' codes for subnational country regions at the highest granularity level (GeoNames Admin 1), which is roughly equivalent to NUTS 1 level in Europe and states and provinces in most other countries such as the US.

Multiple measures could be used to estimate the overall share of scholars who were mobile. The most straightforward measure could be the count of unique researchers with more than one subnational region (internal mobility, 3,078,037 researchers out of 19,050,557, 16.16%) or country of affiliation (international mobility, 1,426,098 researchers, 7.49%) throughout a researcher's publication career. While this measure shows the overall share of scholars who could have been at risk of migration, a more conservative measure could use the more strict "mode-based" method. This is the principal method to identify migration events throughout our manuscript. Table S1 describes the count and percentage of internally and internationally mobile scholars per population and period using the mode-based method. For the period from 1998-2017, the percentage of internally mobile scholars was 8% versus 5% for international mobility. To calculate these percentages, we looked at scholars for which the mode region or country of their institutional affiliation (over a calendar year) changed for at least one year, divided by the unique number of scholars. We used data for the period from 1996-2020. However, the estimates of internal and international migration refer to the period from 1998-2017. The reason for this is that we need some information on the location of scholars before 1998 and after 2017 to assess whether a migration event occurred at the beginning or the end of the period.

Authors with multiple affiliations. The literature shows an increasing trend in the number of authors who have multiple affiliations in their publications (8, 9). Studies using bibliometric data to study migration should clarify how they deal with these cases. Our original database includes authors with multiple affiliations in their publications, and we considered this in our identification of migration events using the mode-based method. Here, we first describe how our mode-based method addresses the issue of multiple affiliations, and further below, we show how prevalent the multiple affiliations are in our database, and what share of authors and identified migration events are affected by it.

Briefly, our approach entails the following steps (and further below more details on each step are provided):

1. We consider all affiliation addresses that are used by an author, either in a single publication, or across multiple publications, over the course of a calendar year.

2. We map all the affiliations to subnational regions.

3. We take the subnational region that appears the largest number of times, for an author during the course of a calendar year (the modal region), as the estimated subnational region of residence for the author during that year.

4. If there is more than one mode, we use all of them, and check whether one of the modes was the previous region of residence, and take that region.

5. If there are gaps in the publication record of the scholar (i.e., no publications for a specific year), we assume that the region of residence of the author remained the same (for up to two years before the year for which a region was identified).

6. When the modal region for the author is different from the one estimated for the previous year, a migration event is recorded.

Addressing multiple affiliations in migration estimation. Tables S2 and S4 show examples of fictitious and actual authorship records, respectively, and how we have structured the database to consider the multiple affiliations used in a paper. For instance, author 1 has three affiliations used in the same paper. Our analysis considers any affiliation address that is used by authors either in a single publication or across multiple publications in a year, as shown in these two tables. In the next step, the mode-based method considers all subnational regions in a year to determine the most occurring one as the mode region of residence, which is shown in Tables S3 and S5, respectively. If there are two or more mode regions in a year, we also consider this and do not reduce it to one mode. A consistent backward filling of two years (considering disciplinary differences in publication practices as documented by (10)) is applied to the years for which we do not have a publication activity but

consider the author still active since a publication was observed in later years and the currently observed publication is a signal for the work that could have been started at least two years earlier (an example is shown for years 2005 and 2006 in Table S2 marked with "filled"). Next, we checked if the mode region or regions include one that is different from the previous year. If that is the case, a migration event is recorded. If the mode region is repeated, no migration event is recorded. For the case of an author who has up to 3 affiliations in the same publication, presented in Table S4 (the publications of only 2019 are shown as examples), we see in Table S5 that due to the repeated presence of the mode regions and overlap between them, no migration events will be recorded from 2015 to 2020. Previously, in (2), we have presented an extensive description of our methods and best practices that were based on prior empirical studies to evaluate different measurement practices and scenarios.

Prevalence of multiple affiliations. To see the prevalence of "multiple affiliations" and how it has changed over time, we need to separate between two types of analysis that are possible using bibliometric data: publication level and author level analysis. Previously, in (11), we have presented how these two approaches could lead to different conclusions as, at the paper level, the trends are rapidly increasing. The author-level analysis clarifies that only a small subset of authors are responsible for the majority of the observed increases. Fig. S1 shows these differences, where on the left, a paper-level picture of all unique publications in a year is presented that includes single or multiple affiliation authors. The trend in multiple affiliations, while present in earlier years, has rapidly increased more recently. However, the right panel in this figure, which is at the author level, shows that the prominent majority of authors have single affiliations. We observed that the overwhelming majority of scholars in the data set have only a single affiliation. The number of authors with a single affiliation only has also increased substantially over time, going from about 1 million unique authors per year in 1996, to above 3 million unique authors in 2019. The number of authors with multiple affiliations increased at a faster pace over time, but the overall size of this group of authors is relatively small. It went from slightly more than 30,000 in 1996 to less than 300,000 in 2019. However, we emphasize that these counts are prone to multiple counting as the same author in a given year could be using multiple affiliations in the next year, which was previously shown in the authorship tables presented above, i.e., Table S4, and is further discussed below.

To further elaborate on these temporal trends, we consider an author's whole publication career and the number of years an author would have single, multiple, or both types of affiliations, presented in Table S6. For 97.7% of 19,050,557 authors in our data, on average, they have 7 years of career with a single affiliation, and the median is 5 years. For 1,375,350 authors (7.22%), which is again prone to multiple counting and hence percentages go beyond 100%, and for an average of 2 years of career (median 1), scholars have multiple affiliations. Please note that the median (50th), 90th, and 99th percentile in the number of unique years are informative here as they show that having multiple or both types of affiliations is rare and occurs for shorter periods over an author's career.

Dominant type of affiliation throughout one's career. Next, we categorized authors based on the most dominant type of affiliation throughout their career, i.e., the highest number of years with a certain type, and took the type with the highest years, presented in Table S7. Here, we see that 96.37% of authors have single affiliations as the dominant type, and for only 2.9% of authors, multiple affiliations occur as the dominant type, and for 0.78% of authors, both types of affiliations are the dominant type.

Effect on migration estimates. To highlight if and to what extent our migration identification results are affected by these multiple affiliation authors, despite the fact that the mode-based method already addresses this to some extent (as discussed above), we investigated the overlap between authors identified as internally and internationally mobile and the ones with multiple affiliations. For internal mobility, based on the mode-based strict method, we have a total of 24,216 unique authors who have had at least one year and publication with "multiple affiliations," and they have been identified in that year as mobile among the total of 1,328,938 authors (from 1996 to 2020), equal to 1.82%. In international mobility, this count is 22,428 out of 847,624 authors, equal to 2.65%.

This empirical investigation clarifies how an author-level analysis of migration events could be less affected by the multiple-affiliations phenomenon. This issue is also discussed in the literature as an increasing trend in terms of publications (8, 9). However, this literature has not considered an author's career-long trajectory in using multiple affiliations versus single affiliations. The studies cited here have only discussed the issue of multiple counting at the publication level analysis due to authors publishing in more than one year while using multiple affiliations. However, this is an increasing phenomenon that should be carefully considered in studies using bibliometric data for migration research.

Additional Measures: Aggregated Net Migration Rate, Migration Effectiveness Index, and Crude Migration Intensity. In addition to the measures presented in the methods section in the main text, to provide a more suitable measure of comparison between multiple countries (12), and to highlight the importance of migration relative to the size of the population of scholars and their subset which has been mobile, we calculated the Aggregated Net Migration Rate (ANMR) as in equation 1. To further complement our analysis and control the effectiveness of the migration events in redistributing the scholars' population between regions inside a country, we calculated the Migration Effectiveness Index (MEI) (12, 13) as in equation 2. Additionally, to evaluate the intensity of internal migration and its trend over time, we computed the Crude Migration Intensity (CMI), as in equation 3.

$$ANMR_t = 100 \times \frac{0.5 \sum_i |I_{i,t,k} - E_{i,t,k}|}{\sum_i N_{i,t}} \quad [1]$$

$$MEI_t = 100 \times \frac{\sum_i |I_{i,t,k} - E_{i,t,k}|}{\sum_i (I_{i,t,k} + E_{i,t,k})} \quad [2]$$

$$CMI_t = 100 \times \frac{\sum_i (I_{i,t} + E_{i,t})}{\sum_i N_{i,t}} \qquad [3]$$

The main difference between ANMR and MEI, as also discussed in (12, 13), is the population of scholars considered in the denominator, which is the total population of scholars in the former and the mobile scholars in the latter. Since the count of scholars in a year could be prone to under- or over-counting, using the count of mobile scholars entering a region or exiting would provide an alternative measure of exposure.

While the net migration rate provides a clear view of the number of scholars (per 1,000) who have entered or exited a region, it does not provide a sufficient basis for comparison between regions in different countries. The Aggregated version (ANMR) and the Migration Effectiveness Index (MEI) are recommended to be used (12) to allow for a better evaluation of how effective migration is in redistributing the population (based on the population at risk in the former case and among the mobile population in the latter case). Another measure is the CMI, which allows for an investigation of the intensity of internal migration relative to the population of scholars in a country. Nevertheless, shortcomings are highlighted (12) that might arise due to the number of regions in each country and the spatial dis-aggregation. We use GeoNames *first* administrative level for all countries to have a consistent view of these countries. However, please note that the CMI could not be compared over different countries due to the same limitation (i.e., the number and size of regions being aggregated is different across countries). However, it is relevant to see the temporal trend of a single country. See Fig. S2 for ANMR, Fig. S3 for MEI, and Fig. S7 for CMI measurements.

Figure S7 shows that in virtually all rich countries, internal migration has been either decreasing or staying relatively stable. This indicates that trends for scholars, a specific group of high-skilled migrants, are not fundamentally different from trends for the whole population, in this respect. In emerging economies and scientific players like India and Brazil, the internal migration of scholars has been increasing. Please note that the CMI cannot be compared across different countries due to different numbers and sizes of subnational regions being aggregated (as discussed above, also see, for more details, (12)). However, the CMI can tell us for each country, whether internal migration has, on average, been increasing or decreasing.

Net Migration Rate trends for the earlier periods 2000-2005, 2006-2011 in comparison with 2012-2017. Our statistical analysis included all observation years from 1998 to 2017 (both inclusive). However, in the maps in Fig. 1 in the manuscript, we selected 2012-2017 as an illustrative example to show the most recent results. Fig. S4 shows the Net Migration Rate for three periods in 2000-2005 (top), 2006-2011 (middle), and 2012-2017 (bottom) to allow for comparison. Additionally, Fig S3 shows these three periods for the Migration Effectiveness Index. Equivalent figures for the whole period, i.e., 1998-2017, are available in our replication materials.

For some subnational regions, the migration rates mostly follow similar patterns as for those in the most recent period. For example, please see the mid-eastern versus the mid-western states in the US. However, the figures for earlier periods also show that, for instance, the predominant part of the outmigration from India happened from 2000 to 2005 and continued at a lower pace from 2006 to 2011, and at an even lower pace from 2012 to 2017. Another illustrative example is Australia, where, from 2000 to 2005, we observed a negative NMR, whereas from 2006 onwards, Australia became, on average, a country of destination for migrant scholars. In contrast, Fig. S3 shows that the MEI, which indicates the rate of turnover of scholars by contrasting those entering or leaving a subnational region over the denominator of all mobile scholars (14), is prone to be affected by the outlier regions (depicted in yellow on the maps). Nonetheless, this measure shows that subnational regions, for instance, in Saudi Arabia in the Middle East, as an illustrative example, which are depicted with lighter colors, indicate a dynamic influx of incoming and outgoing scholars, leading to more changes in the composition of scholars.

Separated net internal and international scholarly migration rates for the world, and over time for the US as an illustrative country. Here we present further results based on net scholarly migration rates at internal and international levels aggregated in subnational regions for the 24 years of observation. Fig. S5 shows the NMR separated over internal and international rates for 2012-2017 (other periods are available in our replication materials). It shows that the pattern of international migration does not necessarily match that of internal migration. Subnational regions have different levels of attractiveness for internal and international migration. For example, while the West Coast in the US is an attractive region for both internal and international scholars, it is not the same case for middle and eastern states. Eastern states are likely more attractive for international scholars than internal ones. In general, many of the states in the mid-eastern area of the US are sending states and have a negative net migration rate (indicated with red colors), and western regions are receiving (green colors). This stark difference between internal and international migration is also observed in provinces in Australia, South America, India, Turkey, Saudi Arabia, Iran, and China. Zooming in on Europe shows even more evident differences. While many provinces in Germany, Italy, France, Spain, the UK, and the Netherlands are indicated with red colors and send scholars to international destinations in the top panel, there is a large difference between internal migration to these provinces in the bottom panel. For instance, the Northern and Central provinces in Italy, Southern regions in France, England in the UK, and some regions in Spain, are receiving scholars from other subnational origins inside these countries. This could signal that, in these regions, academic positions of those leaving for international destinations are filled by internal migrants.

Additionally, we present trends for all states in the USA (see Fig. S6) as one illustrative example of data provided in our database to show how NMR rates change for these states over time. The NMR rates presented further highlight the use cases of our database to identify sending and receiving subnational regions.

Typology of subnational regions based on negative, positive, balanced or mixed NMR. Table S8 presents a typology at the subnational level and categorizes regions based on the sign of NMR to "net receiver" (positive internal (IN) and international (INT) NMR), "net sender" (negative NMR for both measures), "balanced" (both NMRs are 0), and alternating positive or negative NMR, respectively, in IN and INT, and the other way around (we added a "mixed" category for cases that do not strictly follow these patterns e.g., 0 NMR in one measure and positive or negative in the other). In addition, we set the minimum number of years a region has spent in a certain typology to be above 10 years. We selected the typology with the highest number of years as the dominant type for that region. Furthermore, we limit the regions to those with at least 5 years of observation and 200 scholars in the population and show the top 3 regions in each continent (if present). Our replication materials include all these regions and their dominant typology, and one could tweak these thresholds to obtain a different typology. Additionally, we share this typology for all regions that, due to space limitations, were not shown in Table S8.

Table S8 shows that, for instance, some regions in all continents have been consistently a net sender of scholars for above 10 observation years, e.g. Beijing in China, while other regions have been consistently a net receiver of scholars for 10+ years, e.g. Alberta in Canada. These long-term trends can have relevant implications for research and development in these regions, that further research can address in the future.

Disaggregated analysis based on the field of science. There are a number of dimensions that could be explored using our replication materials. Here we list a few that could be used by the scientific community to advance our understanding of migration of scholars. As an illustrative example of the dimensions that can be further pursued in future research, here we provide additional descriptive statistics and figures on migration rates disaggregated by field of science (grouped into three main categories: "Agricultural, Engineering and Technology, and Natural Sciences," "Humanities and Social Sciences," and "Medical and Health Sciences"). Additionally, our database could, in principle, be used to study similar disaggregations by academic age (e.g., years since first publication) and by cumulative number of publications. Here we are not providing such analyses as they would require a more in-depth operationalization of age and productivity to address potential left-censoring issues in the data.

We computed net migration rates disaggregated by field of science for three main periods: 2000-2005, 2006-2011, and 2012-2017. The field assignment for each scientist was done by considering their entire career. It is based on the field classification of all publications by a scholar. Some publications could be classified into multiple fields, e.g. in the case of interdisciplinary publications. In those cased, we take all the disciplinary allocations and divide the number of publications over these fields in the form of a fractional count, i.e. a publication belonging to two fields will be assigned 50% to each field. We then sum these fractions and calculate the proportion of a scholar's publications in each field. Then we take the field with the highest fractional proportion. The fields are categorized as six macro fields, based on the OECD classification (15). To ease visualization and reduce the dimensionality of figures for enhanced interpretation, we grouped the six fields into three broad categories: "Agricultural, Engineering and Technology, and Natural Sciences," "Humanities and Social Sciences," and "Medical and Health Sciences".

Fig. S8 shows net migration rates by field of science for the most recent period (2012-2017). Our replication materials include the data and figures for all periods by field of science. A separate set of figures shows rates for the entire period, instead of periods of six years. These disaggregations by field of science provide additional details about the estimates that we produced. As an illustrative example, consider California. This U.S. state has positive net migration as a whole. However, when we consider fields of science, we observe that, while net migration is strongly positive for "Agricultural, Engineering and Technology, and Natural Sciences," and "Medical and Health Sciences," it is negative for "Humanities and Social Sciences." This observation, and related ones for other geographic regions, are descriptive in nature. While we could speculate on some of the reasons for the observed trends (e.g., for California, the structure of its academic and industry ecosystem may favor technical fields), the goal of these additional disaggregations is to provide new estimates, without fully integrated theoretical explanation, that would spur further analyses and would be a valuable starting point for future research. For India, as another illustrative example, Fig. S8 shows that while in Engineering and Natural Sciences, and Medical and Health Sciences, India has a negative net migration rate for most regions, for Humanities and Social Sciences, it has a highly positive NMR for some regions. This could indicate that some regions receive scholars from abroad, or the return of Indian scholars from abroad, or arrivals from other internal origins.

To compute the Net Migration Rates specific to the "disaggregation" variable that we considered (i.e., field of science), we used the following formula:

$$NMR_{i,t,k,d} = 1000 \times \frac{I_{i,t,k,d} - E_{i,t,k,d}}{N_{i,t,d}} \quad [4]$$

where the definition of variables and indexes are exactly the same as those presented for the Net Migration Rate (NMR) in the manuscript. However, here we have introduced an additional index d, which stands for the "disaggregation" variable used. For fields of science, that would be the three categories of "Agricultural, Engineering and Technology, and Natural," "Humanities and Social," and "Medical and Health Sciences". We took all scholars in that specific category as the population and used it in the denominator, i.e. N. In the nominator, we disaggregated the in-migration (I) and out-migration, (E), in a given year, so that it includes only the specific category of analysis (e.g., only scholars in "Medical and Health Sciences" who have entered this sub-national region or exited the region). This way, we are able to compare scholars with others in the same field of science and consider their in and out migrations, which are then used to calculate NMRs.

Investigating these nuanced disaggregated trends requires further research that is outside the scope of this study, but the replication materials of our study enable these types of future research.

Estimation of subnational migration trends within continents using Generalized Additive Mixed Models (GAMMs). Migration trends were estimated separately for each continent (Fig. S9) using Generalized Additive Mixed Models (GAMMs), accounting for variations in regional trends. GAMMs extend Generalized Linear Mixed Models (GLMMs) by incorporating penalized smooth functions, allowing for the modeling of non-linear relationships between predictor variables. We use a quasi-Poisson distribution (log as the link function) to account for potential over-dispersion and Restricted Maximum Likelihood (fREML) as a fitting method. In GAMM, migration rates were modeled using count data models with offset set to the log number of scholars in a specific region and for a given year (exposures approximation). The model was fitted in R (16) using the *gam*() function of the *mgcv* package (17). We use the following formula:

$$log\ counts \sim s(t) + s_{fs}(t, i) + offset$$

where $s(t)$ is a smooth function of the year t using a p-spline basis and $s_{fs}(t, i)$ is a factor smooth interaction modeling the random effects of region i. We also tested an alternative simpler model:

$$log\ counts \sim s(t) + s_{re}(t, i) + s_{re}(i) + offset$$

where $s_{re}()$ is used to model simple uncorrelated random effects (random slope and random intercept). Both models gave very similar results. Please see (18) for different applications of similar models.

Fig. S9 shows the temporal change in two systems of migration, i.e. internal and international, compared across in-migration and out-migration. In all continents, the in-migration for both international (blue line) and internal (red line) flows stays above the out-migration for international (green line) and internal (yellow line) flows. In Africa and Oceania, the international rates have a large gap with the internal rates and the magnitude of internal migration is much higher, i.e., close to twice the international one. In Europe and North America, the pattern is not as strong: the internal rates are above the international rates, but magnitude is closer and the gap is less pronounced. In most continents, the trends are decreasing over time, meaning that migration of scholars is decreasing (with the exception of internal out-migration in Africa, Asia and South America, and internal in-migration in South America).

Two-stage algorithm for estimating migration trends. Fig. 3 in the manuscript presents the slopes estimated from multiple quasi-Poisson regressions alongside the results of a Bayesian linear model fitted to these slopes.

Rather than fitting a single large interaction model, we adopted a two-stage approach. First, we conducted multiple region-specific regressions. Then, we applied a Bayesian linear regression model to these slopes, incorporating uncertainty in both dependent and independent variables through their standard errors (see next subsection for details). This approach is preferable because high-dimensional interaction models often fail to converge and, as noted by Lewis and Linzer (19), assume an unrealistic absence of residual variation in individual-level coefficients.

Stage 1: Estimating regional trends using quasi-Poisson regressions.
In the first stage, we estimated time trend slopes for each region separately using independent quasi-Poisson regressions. Migration counts served as the dependent variable, modeled with a log link function and an offset term for the log number of scholars in a given region and year, allowing us to model a rate by accounting for varying numbers of scholars. The quasi-Poisson model was chosen to address overdispersion, a common issue in count data that can lead to underestimated standard errors in standard Poisson regression.

The model for each region can be written as:

$$\log(\lambda_{it}) = \beta_0 + \beta_1 \cdot t + \log(\mathrm{E}_{it}) + \epsilon_{it}$$

Where:

- λ_{it} is the expected number of migrations in region i at year t,
- β_0 is the intercept,
- β_1 is the year trend slope,
- t is the year,
- E_{it} is the number of scholars in region i at year t (offset term),
- ϵ_{it} is the error term.

Stage 2: Bayesian linear regression to model slope uncertainty.
The second stage employed a Bayesian linear model fitted to the estimated slopes. This approach accounts for varying standard errors in both dependent and independent variables. The model was implemented using the `brms` R package (20, 21). The `brms` package allows modeling of measurement error in predictors via the `me()` function, specified as $\mathrm{me}(x, \mathrm{se\_x})$, where se_x represents the standard error of the predictor variable. Measurement error in the response variable can be handled using two methods:

- The `se()` method explicitly incorporates measurement error using standard errors:

$$y \mid \mathrm{se}(\mathrm{se\_y}, \sigma = \mathrm{TRUE}) \sim 1 + \mathrm{me}(x, \mathrm{se\_x}) \qquad \text{[se() method]}$$

  where se_y represents the standard error of the response variable.

- The `mi()` method treats measurement error as a missing data problem, using Bayesian imputation:

$$y \mid \text{mi}(\text{se\_y}) \sim 1 + \text{me}(x, \text{se\_x}) \qquad \text{[mi() method]}$$

In this paper, we present results obtained using the `se()` approach, but both methods yield nearly identical results.

Interpretation of results.

The results reveal substantial heterogeneity in temporal migration rate changes across regions (Fig. 3 in the manuscript). While trends fluctuate significantly in some regions, such as Africa and Asia, they remain relatively stable in others, like North America and Oceania. For instance, in Asia, international out-migration rates range from a 20% annual decline to a 30% increase, with the most extreme cases occurring in regions with fewer scholars, where statistical uncertainty is higher (blue points in Fig. 3 in the manuscript). Though individual annual changes may appear small, their cumulative effect over time can lead to significant long-term shifts and growing inequalities. For example, a 10% annual decline in migration rates from an initial 100 scholars per 1000 would reduce this figure to 59 per 1000 after five years: $100 \times (1 - 0.1)^5$.

The impact of proportional changes depends on baseline migration rates and population sizes. Even small percentage declines can result in substantial absolute losses in regions with high baseline migration rates or large populations. For example, a 50% decrease in a small research community may reduce the number of scholars from 30 to 15—significant in relative terms but modest in absolute numbers. However, in a large research-intensive region, the same proportional decline (e.g., from 400 to 200 scholars) represents a major absolute loss, potentially disrupting institutional stability, research output, and international collaboration networks.

Interpreting these temporal slopes requires careful consideration of regional contexts. In large research hubs with high baseline migration rates, even slight temporal declines can have meaningful demographic and policy implications. Conversely, regions with lower baseline rates, such as Africa, may experience large relative fluctuations that appear dramatic but have less pronounced absolute impacts.

Kendall rank coefficients for in- and out-migration at subnational region level. The Kendall rank coefficient (22) was used to measure the ordinal association between two variables and it is based on ranking the elements of the sample. In Fig. S10 and S11, we calculated Kendall rank coefficients independently for each regional trend.

Trends of internal and international migration based on World Bank income groups. In addition to Fig. 3 in the manuscript, which showed subnational regions grouped on the basis of geographical macroregions (i.e. continents), here we group subnational regions based on a variable that is less driven by geography and more by development levels. For this, we selected the level of income (based on the World Bank categories) and considered low, middle, upper-middle, and high-income countries. Fig. S12 clarifies that while a more in-depth investigation of other factors associated with scholarly migration is necessary, the level of development of a country, which is also previously shown by us at the country level (4), is highly associated with the presented trends. For instance, subnational regions in Africa show trends that match those in lower-income contexts. In contrast, subnational regions in Europe, North America, and Oceania, in geographic terms, show trends that match the upper-middle and high-income context. Our replication materials enable further research that could investigate the association between subnational-level economic development and scholarly migration.

Data Availability. All scripts, data, and materials to replicate our analysis and figures are deposited in the permanent repository available at this link https://doi.org/10.5281/zenodo.15047101(23).

Table S1. Count and percentage of internally (IN) and internationally (INT) mobile scholars (based on mode-based strict method) over the population of scholars by two periods.

Period	IN mobile	IN %	INT mobile	INT %	Population	Publications
1998-2017	1,177,104	7.74	755,072	4.97	15,207,423	24,412,099
1996-2020	1,328,938	6.98	847,624	4.45	19,050,557	30,757,444

Table S2. An illustrative example of authorship records for a fictitious author.

Author name	Publication ID	Affiliation	Region	Year
Author 1	Paper 1	Affiliation A	Region A	2001
Author 1	Paper 1	Affiliation B	Region B	2001
Author 1	Paper 1	Affiliation C	Region C	2001
Author 1	Paper 2	Affiliation A	Region A	2001
Author 1	Paper 2	Affiliation C	Region C	2001
Author 1	Paper 3	Affiliation A	Region A	2002
Author 1	Paper 4	Affiliation C	Region C	2003
Author 1	Paper (filled)	Affiliation D (filled)	Region D	2005
Author 1	Paper (filled)	Affiliation D (filled)	Region D	2006
Author 1	Paper 5	Affiliation D	Region D	2007
Author 1	Paper 6	Affiliation C	Region C	2008
Author 1	Paper 7	Affiliation E	Region E	2009
Author 1	Paper 8	Affiliation E	Region E	2010

Table S3. An illustrative example of how the mode-based method transforms the authorship records in Table S2 for a fictitious author to mode region of residence per year.

Author name	Year	Mode region
Author 1	2001	[‘Region A’ ‘Region C’]
Author 1	2002	Region A
Author 1	2003	Region C
Author 1	2005	Region D
Author 1	2006	Region D
Author 1	2007	Region D
Author 1	2008	Region C
Author 1	2009	Region E
Author 1	2010	Region E

Table S4. An illustrative example of actual authorship records for an author in Scopus with multiple affiliations. The table is limited to only 2019 to maintain clarity. IDs are masked to maintain anonymity and compliance with the Scopus license terms.

Author ID	Publication ID	Year	Region
Author X	Publication 1	2019	FI.01
Author X	Publication 1	2019	DE.12
Author X	Publication 1	2019	GB.ENG
Author X	Publication 2	2019	FI.01
Author X	Publication 2	2019	GB.ENG
Author X	Publication 2	2019	DE.12
Author X	Publication 3	2019	DE.12
Author X	Publication 3	2019	GB.ENG
Author X	Publication 3	2019	FI.01
Author X	Publication 4	2019	FI.01
Author X	Publication 4	2019	GB.ENG
Author X	Publication 5	2019	DE.12
Author X	Publication 5	2019	FI.01
Author X	Publication 5	2019	GB.ENG
Author X	Publication 6	2019	DE.12
Author X	Publication 6	2019	FI.01
Author X	Publication 6	2019	GB.ENG
Author X	Publication 7	2019	DE.12
Author X	Publication 8	2019	FI.01
Author X	Publication 8	2019	DE.12
Author X	Publication 8	2019	GB.ENG
Author X	Publication 9	2019	DE.12
Author X	Publication 9	2019	FI.01
Author X	Publication 9	2019	GB.ENG

Table S5. An illustrative example of how the mode-based method transforms the authorship records in Table S4 for the selected author to mode region of residence per year across all years of career. In the year 2019, shown in Table S4, this author has 3 regions identified as mode.

Author ID	Year	Mode region
Author X	2009	[‘DE.12’ ‘US.PA’]
Author X	2010	DE.12
Author X	2011	DE.12
Author X	2012	DE.12
Author X	2013	DE.12
Author X	2014	GB.ENG
Author X	2015	DE.12
Author X	2016	[‘DE.12’ ‘FI.01’ ‘GB.ENG’]
Author X	2017	DE.12
Author X	2018	[‘DE.12’ ‘GB.ENG’]
Author X	2019	[‘DE.12’ ‘FI.01’ ‘GB.ENG’]
Author X	2020	DE.12

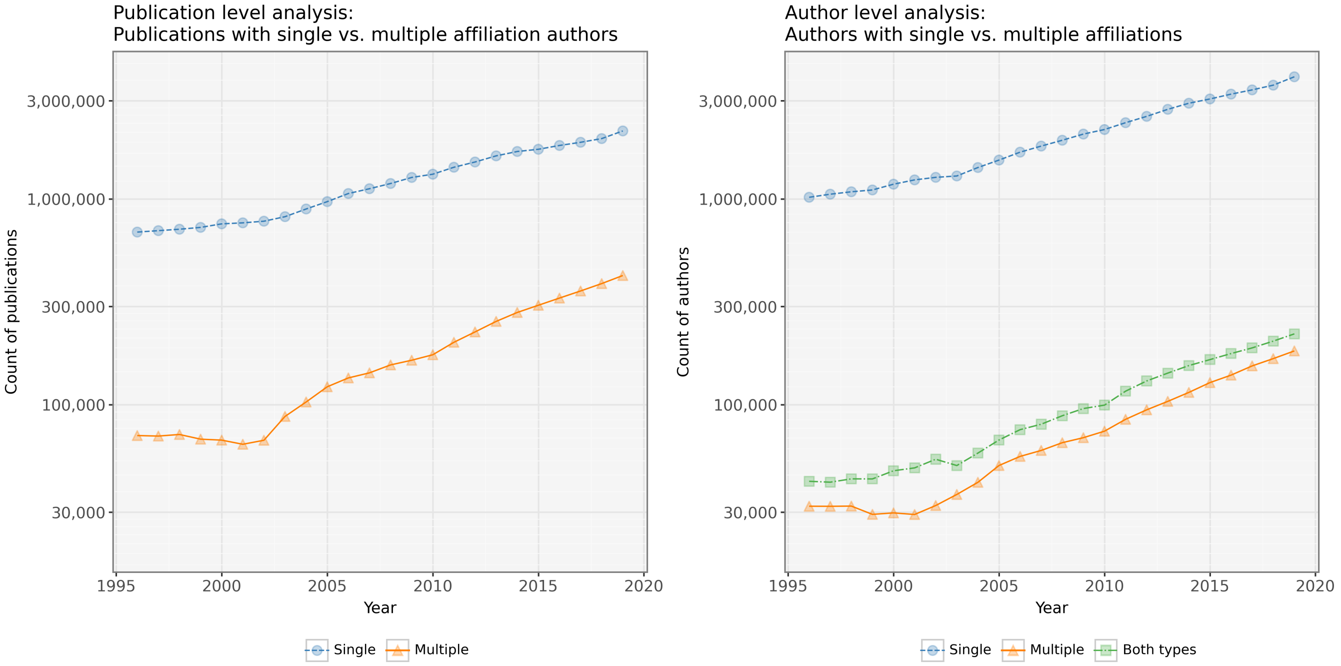

Fig. S1. The difference between publication level (left) and author level (throughout career, right) analysis of multiple affiliations. Please note that counts in both plots are based on multiple counting of authors who continue publishing in following years, and Tables S6 and S7 provide further details on the unique count of authors.

Table S6. Count and percentage of authors who have had multiple affiliations throughout their career and the descriptive characteristics of the number of years spent in each category (e.g., average years, standard deviation, minimum, maximum, 1%, 10%, etc.). Please note that percentages are calculated based on the unique count of authors, i.e., 19,050,557; since categories include multiple counting, these go beyond 100%.

Affiliation	Authors in category (%)	mean years	std	min	1%	10%	25%	50%	75%	90%	95%	99%	max
Both types	1,160,938 (6.09%)	4	4	1	1	1	1	3	6	10	14	19	25
Multiple	1,375,350 (7.22%)	2	2	1	1	1	1	1	3	4	6	9	20
Single	18,603,987 (97.65%)	7	6	1	1	1	2	5	11	17	20	23	25

Table S7. Count and percentage of authors based on the dominant typology of affiliation throughout their career, i.e., the type of affiliation that the author has held for the highest number of years. One dominant typology is assigned to each author. Please note that percentages are calculated based on the unique count of authors i.e., 19,050,557.

Affiliation	N unique authors	Percent
Both types	147,815	0.78%
Multiple	543,201	2.85%
Single	18,359,541	96.37%

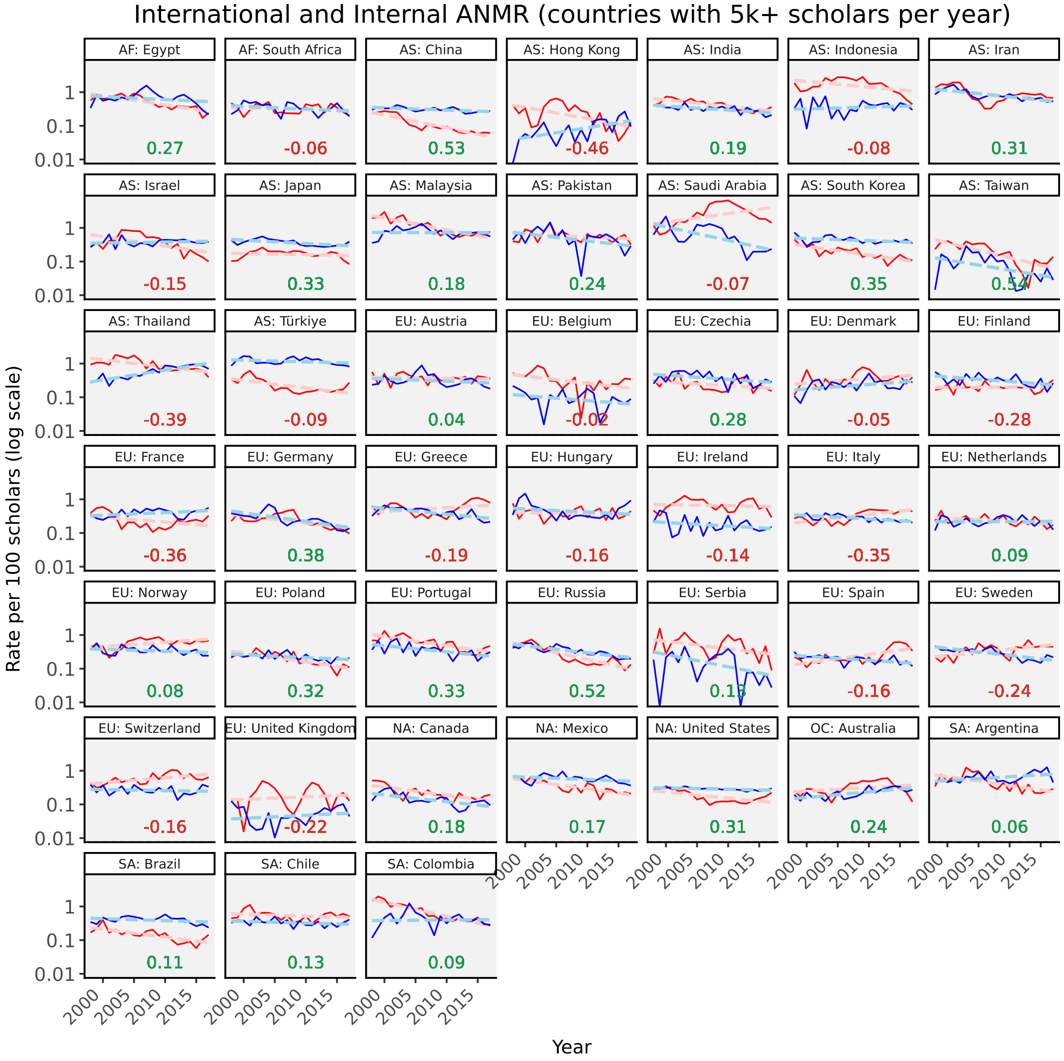

Fig. S2. Countries with more than five thousand scholars per year and internal (blue, dashed line with a lighter color for the smooth trend using LOESS function based on mean) and international (red) scholarly mobility and its comparative and aggregate view in terms of aggregate net migration rate (i.e., ANMR) per 100 scholars. Numbers printed in the bottom part of each panel are Kendall correlation of the internal and international ANMR over the 24 years of observation indicated in green (positive correlation) or red (negative correlation). This figure provides a nuanced *temporal* view of the continent (panel labels) and country differences in ANMR measure where some countries dominate internal and some international scholarly migration that is stable over 24 years of observation, but in general, most countries have declining or stable scholarly migration. 13 countries, most of which in the EU had negative correlations between two migrations.

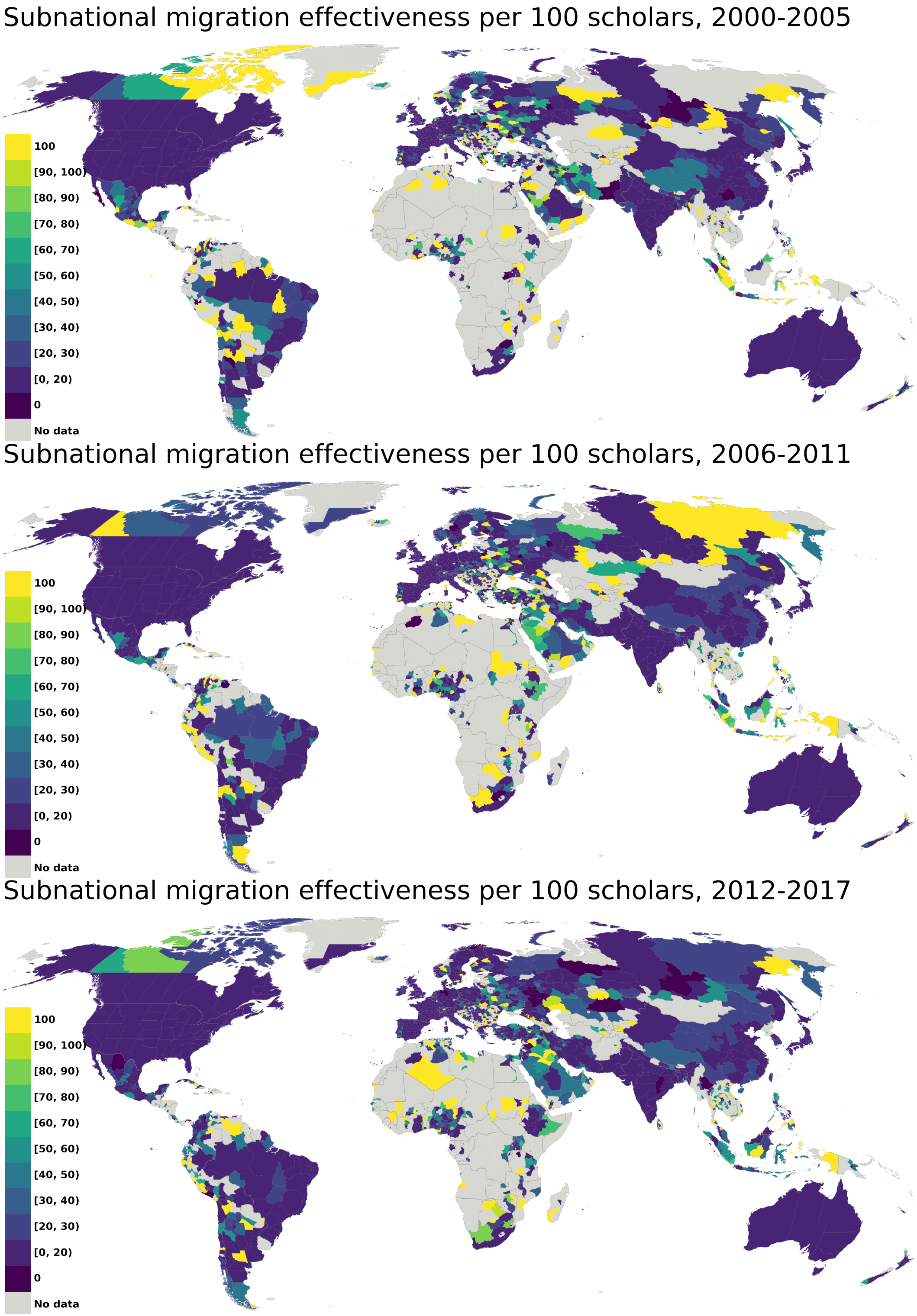

Fig. S3. Subnational migration effectiveness index per 100 scholars in 2000-2005 (top), 2006-2011 (middle), and 2012-2017 (bottom). MEI ranges from 0 to 100 and indicates the overall rate of movements. Lighter and yellower colors show the regions with higher fluctuating rates of movement and darker and bluer colors show regions with more stable migration flows.

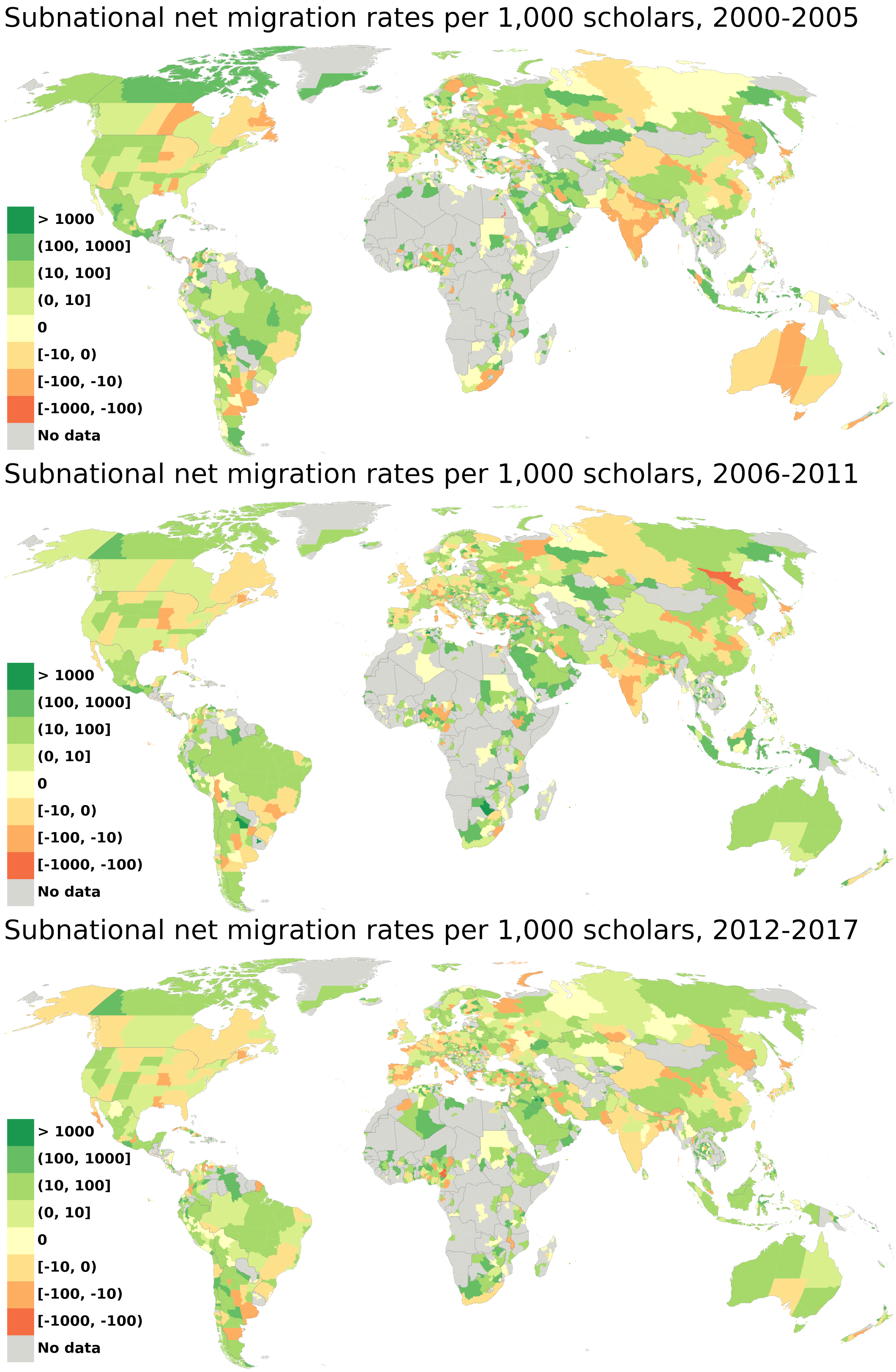

Fig. S4. Subnational net migration rates per 1,000 scholars for the periods from 2000-2005 (top panel), 2006-2011 (middle panel), and 2012-2017 (bottom panel).

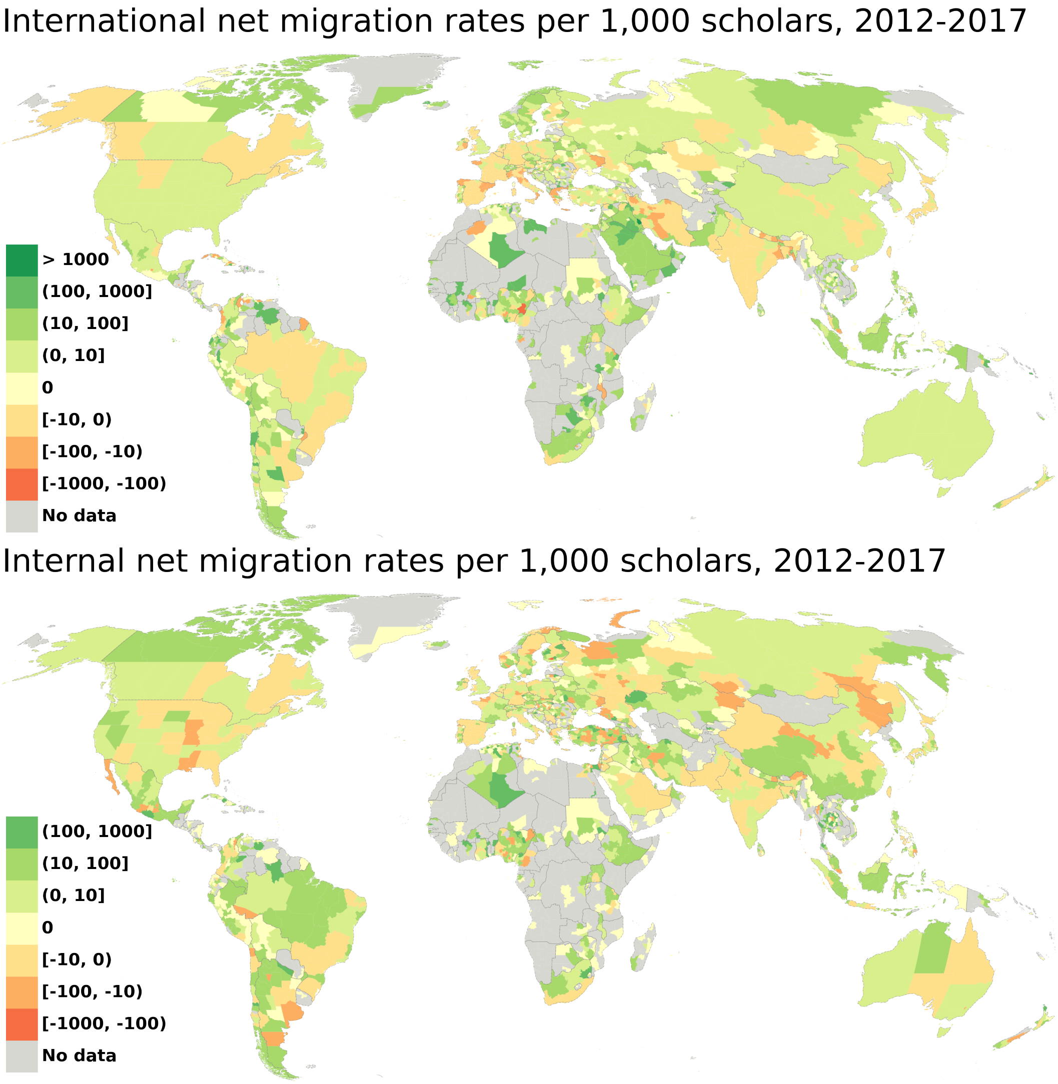

Fig. S5. Separated international (*top*) and internal (*bottom*) net migration rate per 1000 scholars at the province level (see Fig. 1 in the main text for the combined version). Colors present the net migration rate of scholars sent (negative, red colors) or received (positive, green colors), and yellow shows a balanced flow. Color scales are kept similar in all maps to allow comparison. Numbers printed on the legend are the net rate of scholars sent or received from/to international origins/destinations (top) or internal ones (bottom).

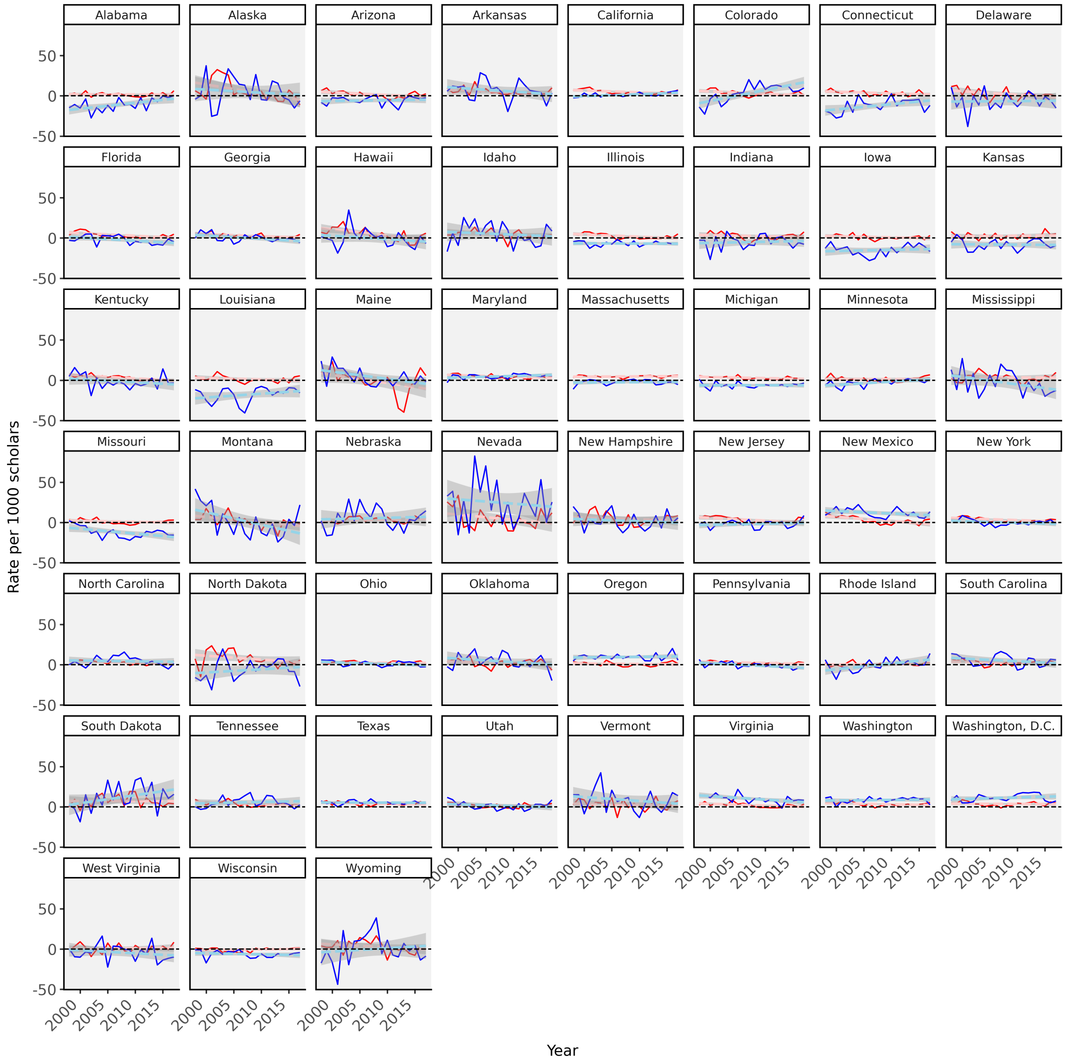

Fig. S6. Net internal (blue) and international (red) scholarly migration rates of US states (dashed lines are smooth trends based on mean). The first observation is the larger range of internal scholarly mobility versus international one (that causes the Y axis of the figure to have a longer range, i.e., -50 to 100). Some states are *sending* states in internal mobility (such as Alabama, Connecticut, Delaware, Iowa, Kansas, Louisiana, and Missouri), while some are *receiving* states (such as Colorado, Nevada, New Mexico, Oregon, South Dakota, Virginia, Washington, and Washington, D.C.). In international mobility, most regions are close to the zero line (which is mainly due to the higher range of internal mobility causing these trends to be less clear) except Alaska, North Dakota and South Dakota which are *receiving* states. Nevada has a high variation in both internal and international scholarly mobility. (Note that seven states out of 50 are missing from the figure as they did not have representation in our results).

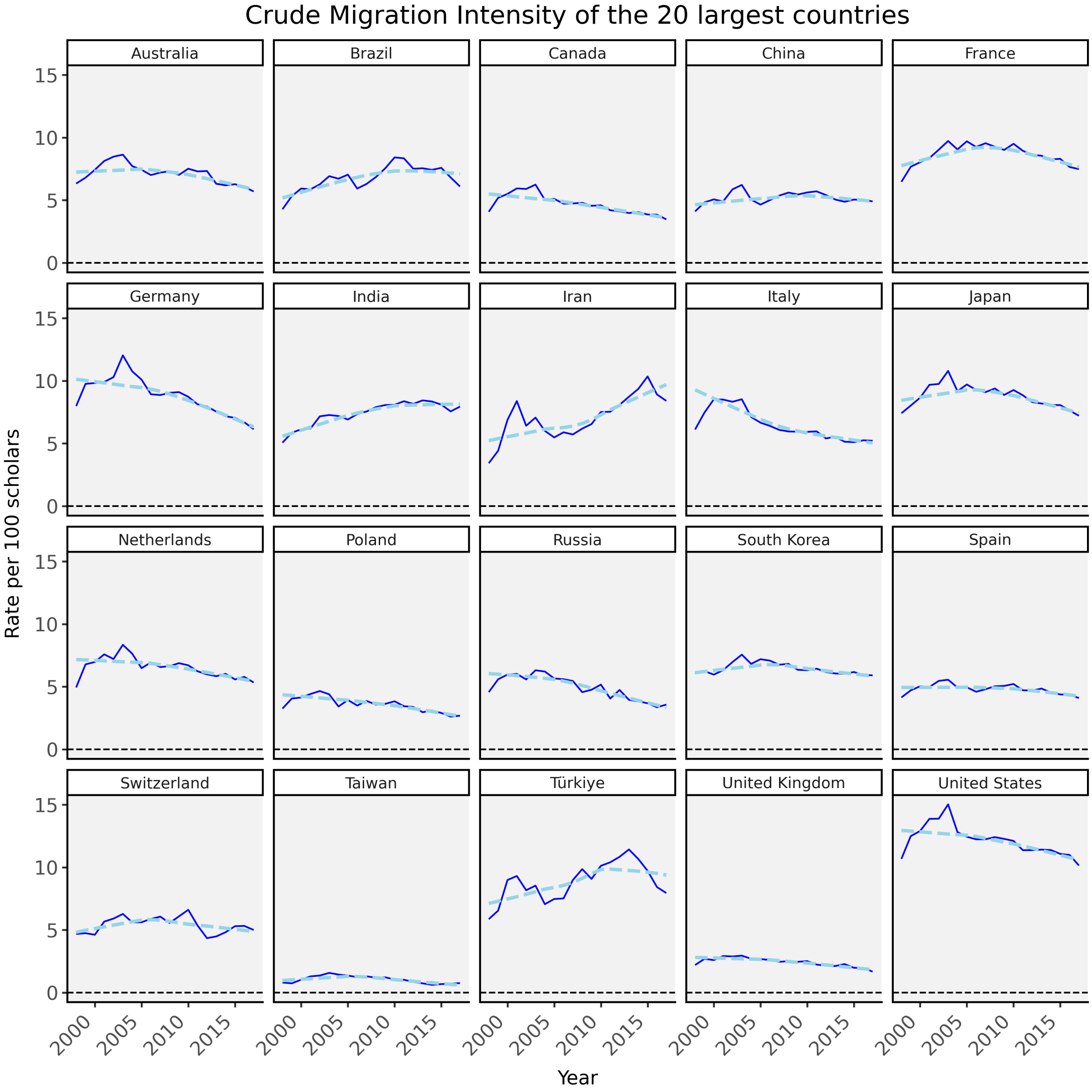

Fig. S7. Crude internal migration intensity rates per 100 scholars for the 20 countries with the largest population of scholars (dashed lines are smooth trends based on mean). Please note that CMI could not be compared over different countries due to different numbers of regions being aggregated (see more in (12)).

Table S8. Typology of the first three subnational regions per continent based on positive, negative, balanced, and mixed NMRs (IN = internal, INT = international, rec. = receiving, sen. = sending, N = count, y = years, "N. y. in typ." = number of years spent in the dominant NMR typology. "N. obs. y." = Total number of observation years. "IN rec. INT sen." = Internal receiver and international sender.). The population is reported for the latest year of the dominant NMR typology.

Typology	Continent	Country	Region name	N. y. in typ.	N. obs. y.	Population
IN rec. INT sen.	Africa	Egypt	Cairo	10	25	1,150
IN rec. INT sen.	Asia	Bangladesh	Dhaka	11	25	2,822
IN rec. INT sen.	Asia	India	Gujarat	10	25	3,497
IN rec. INT sen.	Asia	India	Uttarakhand	11	25	784
IN rec. INT sen.	Europe	Belgium	Wallonia	13	25	4,005
IN rec. INT sen.	Europe	Finland	Pirkanmaa	10	25	1,961
IN rec. INT sen.	Europe	Finland	Uusimaa	11	25	5,180
IN rec. INT sen.	North America	Canada	British Columbia	10	25	13,464
IN rec. INT sen.	North America	Canada	Nova Scotia	10	25	2,601
IN rec. INT sen.	North America	Canada	Ontario	12	25	44,261
IN rec. INT sen.	South America	Argentina	Santa Fe	11	25	1,564
IN rec. INT sen.	South America	Brazil	Federal District	10	25	2,813
IN rec. INT sen.	South America	Brazil	Pernambuco	10	25	4,091
INT rec. IN sen.	Africa	Morocco	Marrakesh-Safi	11	25	555
INT rec. IN sen.	Africa	South Africa	KwaZulu-Natal	13	25	1,105
INT rec. IN sen.	Africa	Tunisia	Safaqis	12	25	960
INT rec. IN sen.	Asia	China	Liaoning	12	25	24,371
INT rec. IN sen.	Asia	Indonesia	West Java	16	25	277
INT rec. IN sen.	Asia	Jordan	Amman	10	25	972
INT rec. IN sen.	Europe	Czechia	South Moravian	12	25	5,143
INT rec. IN sen.	Europe	Denmark	Central Jutland	10	25	4,609
INT rec. IN sen.	Europe	Denmark	Capital Region	18	25	5,943
INT rec. IN sen.	North America	Canada	Manitoba	11	25	1,481
INT rec. IN sen.	North America	Mexico	Puebla	10	25	589
INT rec. IN sen.	North America	Mexico	State of Mexico	10	25	476
INT rec. IN sen.	Oceania	Australia	ACT	11	25	8,051
INT rec. IN sen.	South America	Brazil	Minas Gerais	11	25	2,459
INT rec. IN sen.	South America	Chile	Biobio	10	25	882
Mixed	Africa	Algeria	Algiers	10	25	834
Mixed	Africa	Algeria	Annaba	10	25	620
Mixed	Africa	Algeria	Biskra	12	25	260
Mixed	Asia	Armenia	Yerevan	11	25	268
Mixed	Asia	Azerbaijan	Baki	17	25	237
Mixed	Asia	Georgia	T'bilisi	16	25	549
Mixed	Europe	Bosnia and Herzegovina	Federation of B&H	22	25	597
Mixed	Europe	Czechia	Liberecky kraj	11	25	317
Mixed	Europe	Iceland	Capital Region	12	25	326
Mixed	North America	Belize	Belize	23	25	496
Mixed	North America	Cuba	Havana	13	25	598
Mixed	North America	Mexico	Yucatan	10	25	240
Mixed	Oceania	American Samoa	Eastern District	24	25	2,205
Mixed	South America	Brazil	Mato Grosso do Sul	11	25	230
Mixed	South America	Brazil	Acre	11	24	217
Mixed	South America	Colombia	Atlantico	10	25	812
Net receiver	Africa	Egypt	Kafr el-Sheikh	11	25	253
Net receiver	Africa	Ghana	Greater Accra	10	25	973
Net receiver	Africa	Nigeria	Kano	10	25	213
Net receiver	Asia	China	Inner Mongolia	10	25	438
Net receiver	Asia	China	Hainan	10	25	2,118
Net receiver	Asia	China	Sichuan	11	25	10,763
Net receiver	Europe	Austria	Salzburg	12	25	829
Net receiver	Europe	Austria	Lower Austria	16	25	553
Net receiver	Europe	Croatia	Osjecko-Baranjska	12	25	420
Net receiver	North America	Canada	Alberta	10	25	14,219
Net receiver	North America	Mexico	Nuevo Leon	10	25	1,311
Net receiver	North America	Mexico	Hidalgo	11	25	316

Table S8. Typology of the first three subnational regions per continent based on positive, negative, balanced, and mixed NMRs (IN = internal, INT = international, rec. = receiving, sen. = sending, N = count, y = years, "N. y. in typ." = number of years spent in the dominant NMR typology. "N. obs. y." = Total number of observation years. "IN rec. INT sen." = Internal receiver and international sender.). The population is reported for the latest year of the dominant NMR typology.

Typology	Continent	Country	Region name	N. y. in typ.	N. obs. y.	Population
Net receiver	Oceania	Australia	Queensland	10	25	7,706
Net receiver	Oceania	Australia	Western Australia	11	25	6,289
Net receiver	Oceania	Australia	New South Wales	13	25	17,609
Net receiver	South America	Brazil	Alagoas	10	25	316
Net receiver	South America	Brazil	Para	10	25	1,403
Net receiver	South America	Brazil	Bahia	13	25	235
Net sender	Africa	Cameroon	Centre	13	25	219
Net sender	Africa	Egypt	Giza	11	25	2,771
Net sender	Africa	Egypt	Dakahlia	13	25	1,077
Net sender	Asia	China	Hunan	12	25	16,446
Net sender	Asia	China	Beijing	12	25	83,013
Net sender	Asia	China	Tianjin	13	25	1,806
Net sender	Europe	Austria	Styria	12	25	1,607
Net sender	Europe	Austria	Tyrol	15	25	953
Net sender	Europe	Belgium	Flanders	10	25	5,089
Net sender	North America	Canada	Quebec	19	25	10,371
Net sender	North America	Mexico	Mexico City	14	25	20,340
Net sender	Oceania	Australia	South Australia	10	25	2,798
Net sender	Oceania	New Zealand	Canterbury	10	25	1,895
Net sender	Oceania	New Zealand	Otago	12	25	1,201
Net sender	South America	Argentina	Buenos Aires	16	25	957
Net sender	South America	Argentina	Cordoba	17	25	1,004
Net sender	South America	Argentina	Buenos Aires F.D.	19	25	2,734

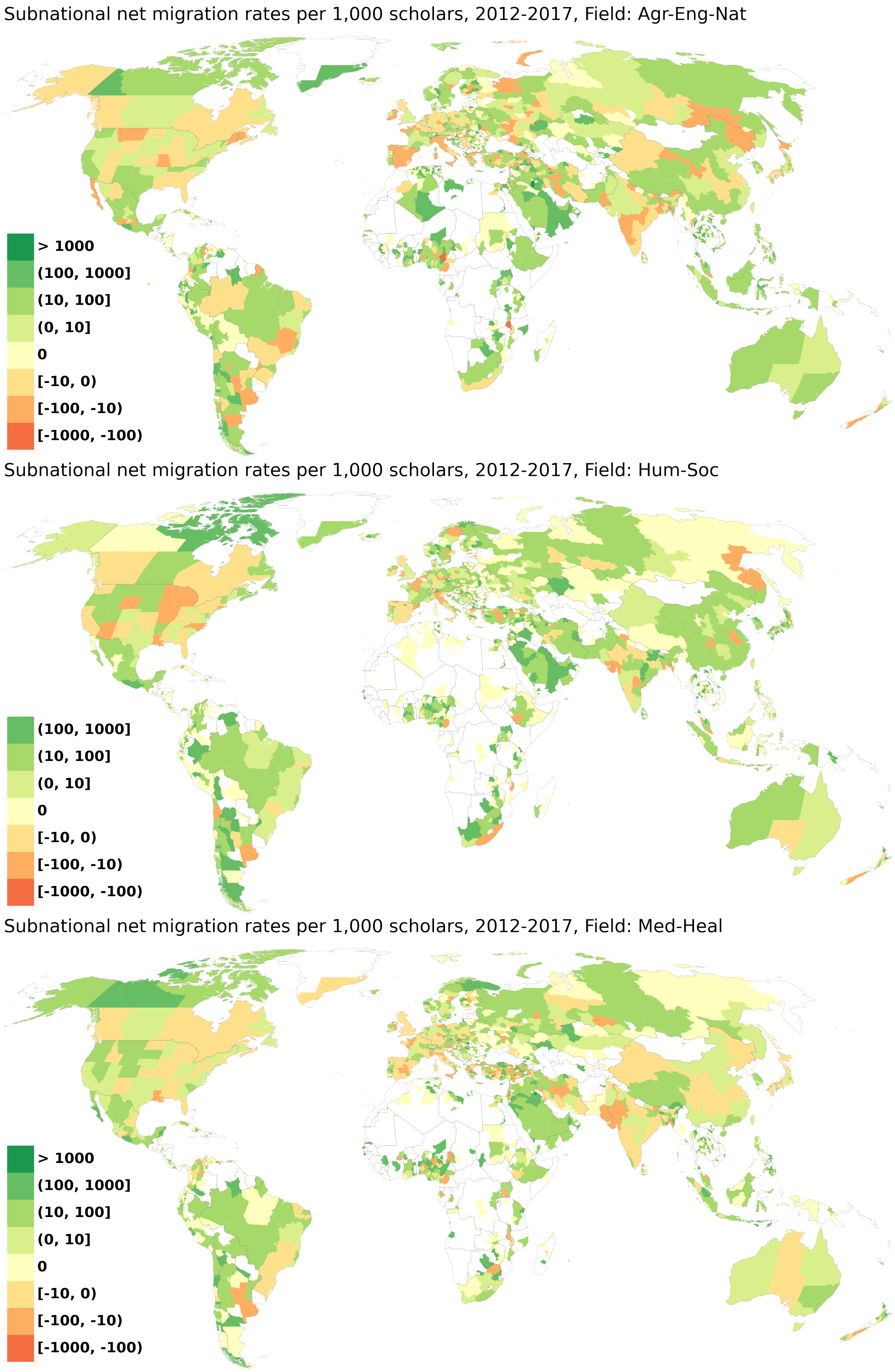

Fig. S8. Subnational net migration rates, per 1,000 scholars, disaggregated by fields of science: "Agricultural, Engineering and Technology, and Natural Sciences" (top panel), "Humanities and Social Sciences" (middle panel), and "Medical and Health Sciences" (bottom panel). Fields of science are based on the OECD classification (15).

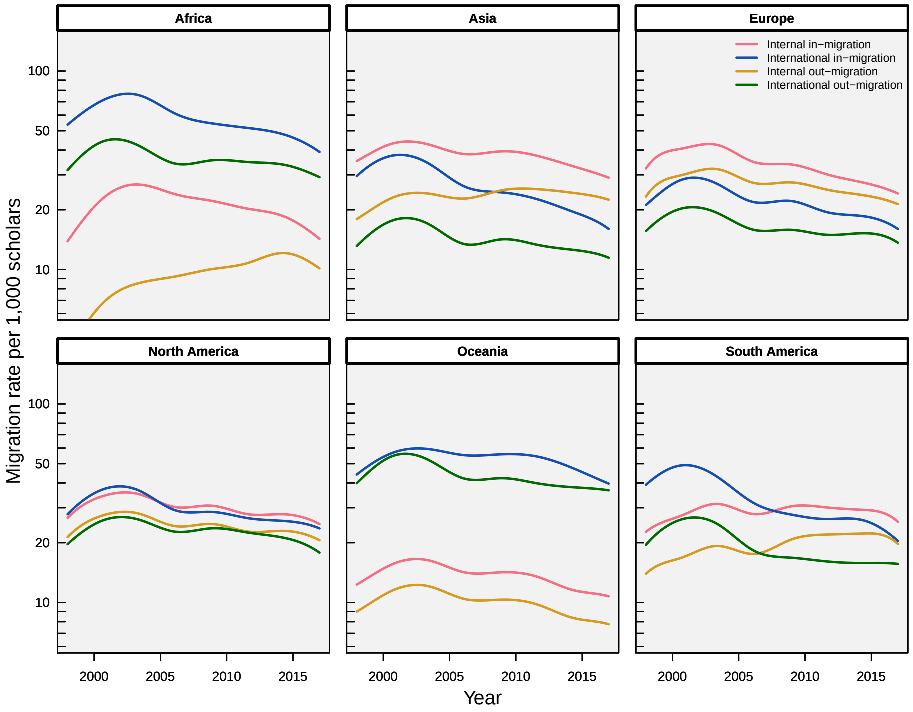

Fig. S9. Predicted migration rates conditional on random effects for different types of migration, year, and continent, estimated via separate GAMMs indicated with line colors. Each line represent an independent model visualized together to show temporal trends. While random effects of regions are included in models, however, they are excluded from the model predictions.

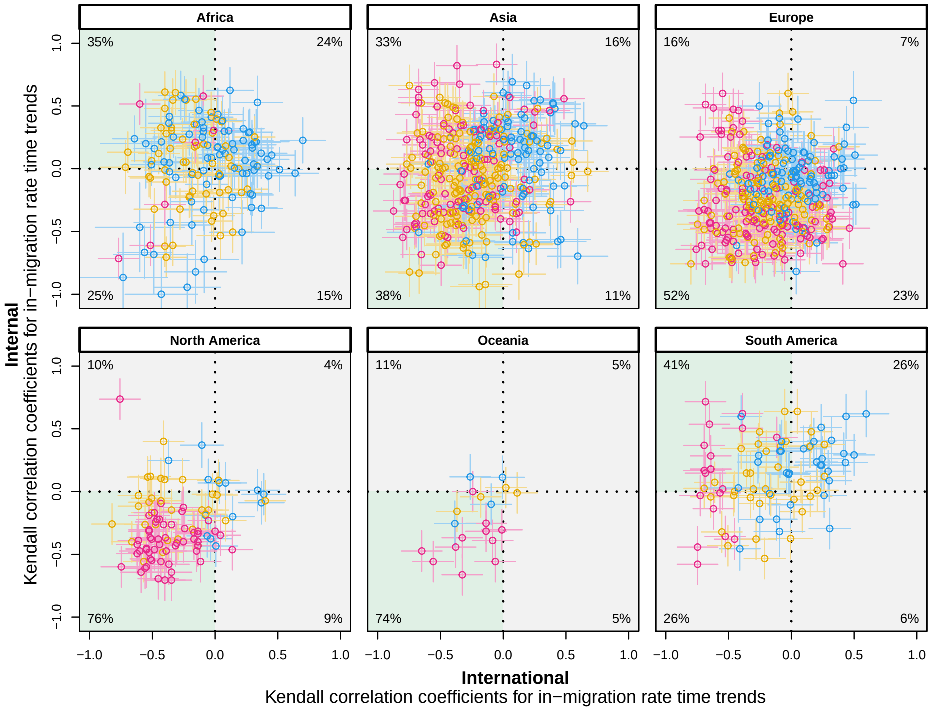

Fig. S10. Kendall correlation coefficients for in-migration rate time trends for different regions (circles) split by continents (6 panels). Blue circles show subnational regions with 0–100 population of scholars, Orange 100–1,000, and Magenta above 1,000. Percent values in each corner denote a fraction of cases per each quadrant. Upper left quadrant: international in-migration rate is negatively, but internal in-migration rate is positively correlated with year; Upper-right quadrant: both international and internal in-migration rates are positively correlated with year; Bottom-left quadrant: both international and internal in-migration rates are negatively correlated with year; Bottom-right quadrant: international in-migration rate is positively, but internal in-migration rate is negatively correlated with year.

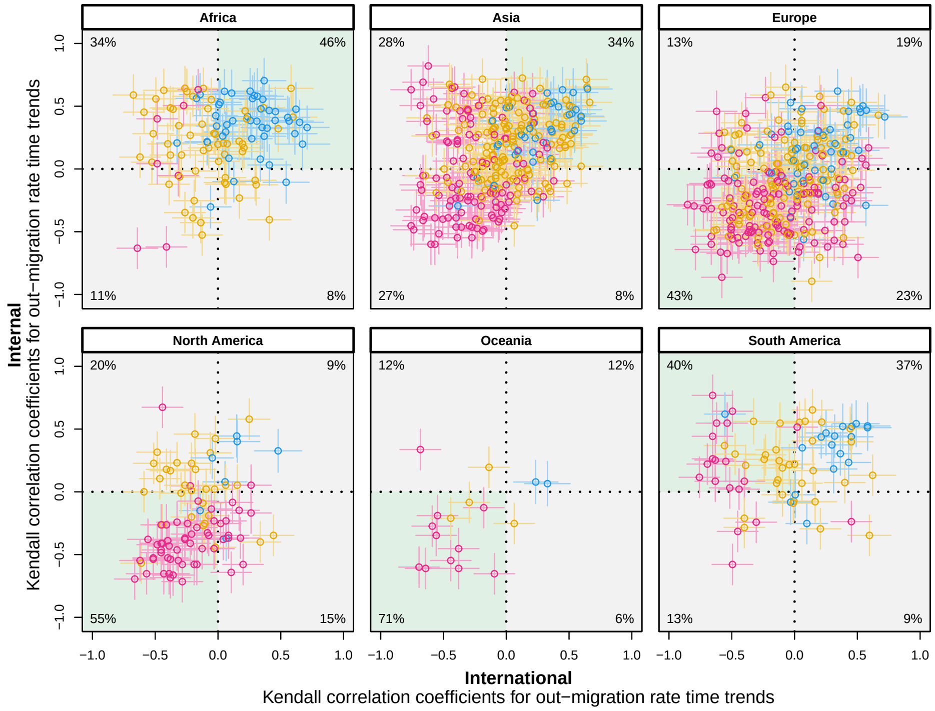

Fig. S11. Kendall correlation coefficients for out-migration rate time trends for different regions (circles) separated by continents (6 panels). The description of this figure is analogous to Fig. S10

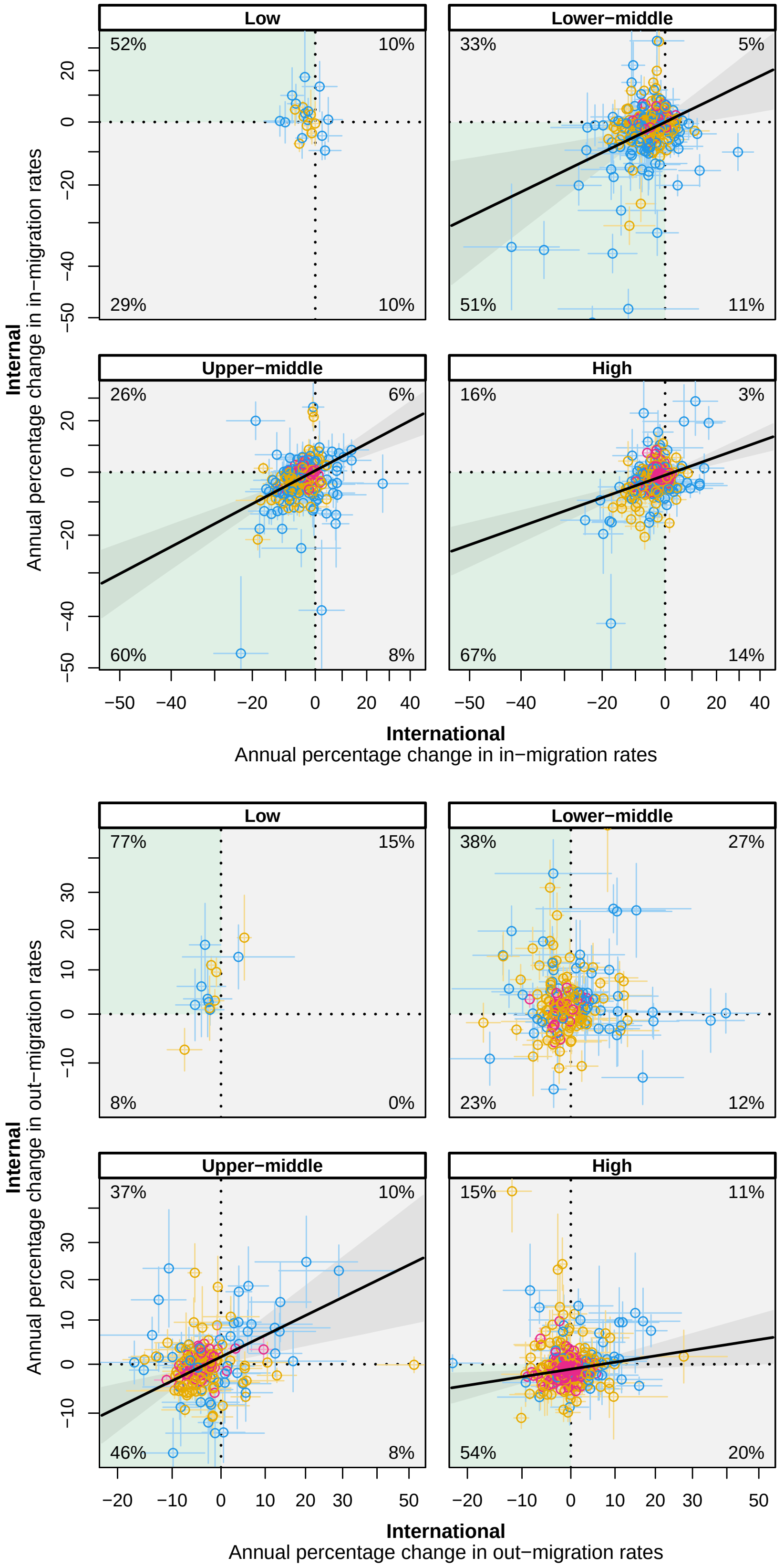

Fig. S12. Complementary visualization for the manuscript's Fig. 3 for slopes of quasi-Poisson regressions of immigration rates (top) and for emigration (bottom) using World Bank's income levels. Labels on X and Y labels show exponentiated slopes indicating percentage increase (positive values) or decrease (negative values) to facilitate interpretation. Blue shows subnational regions with 0–100 population of scholars, Orange 100–1,000, and Magenta above 1,000.

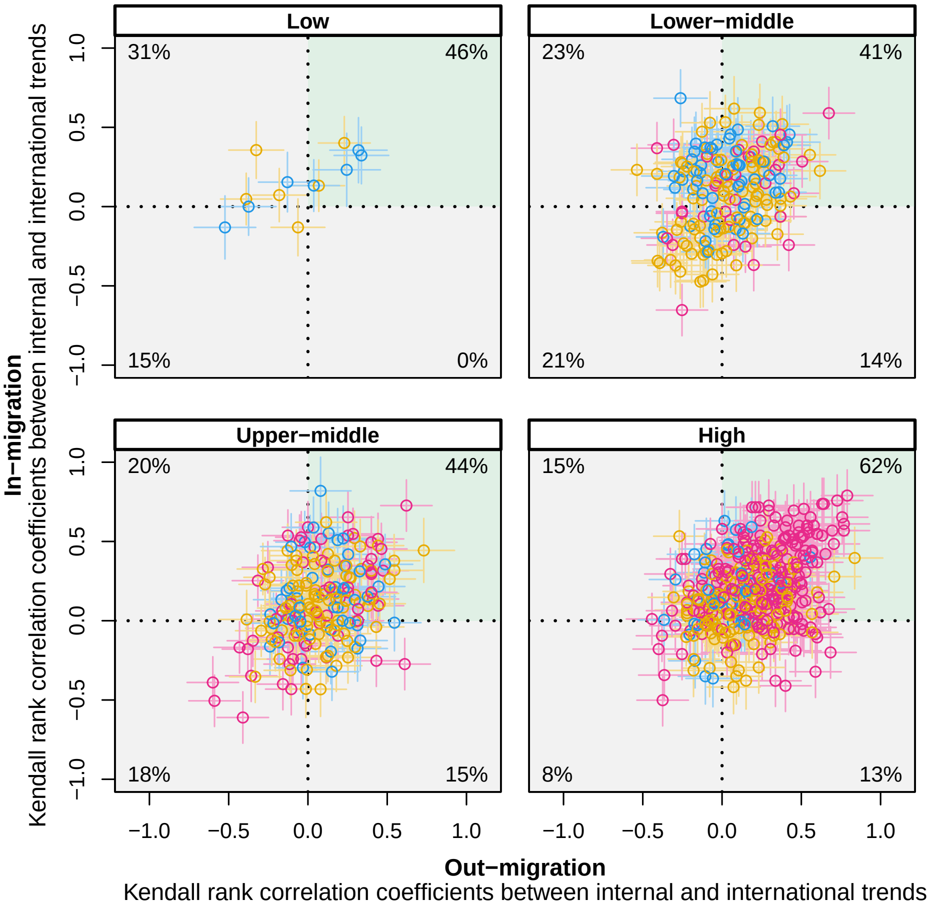

Fig. S13. Complementary visualization for the manuscript's Fig. 4 for Kendall rank correlation between internal and international migration using World Bank's income levels. Blue shows subnational regions with 0–100 population of scholars, Orange 100–1,000, and Magenta above 1,000.

References

1. M Schmidt, et al., The Data Infrastructure of the German Kompetenznetzwerk Bibliometrie: An Enabling Intermediary between Raw Data and Analysis (2024).
2. A Akbaritabar, T Theile, E Zagheni, Bilateral flows and rates of international migration of scholars for 210 countries for the period 1998-2020. *Sci. Data* **11**, 816 (2024).
3. X Zhao, A Akbaritabar, R Kashyap, E Zagheni, A gender perspective on the global migration of scholars. *Proc. Natl. Acad. Sci.* **120**, e2214664120 (2023).
4. E Sanliturk, E Zagheni, MJ Dańko, T Theile, A Akbaritabar, Global patterns of migration of scholars with economic development. *Proc. Natl. Acad. Sci.* **120**, e2217937120 (2023).
5. P Donner, C Rimmert, NJ van Eck, Comparing institutional-level bibliometric research performance indicator values based on different affiliation disambiguation systems. *Quant. Sci. Stud.* **1**, 150–170 (2019).
6. J Baas, M Schotten, A Plume, G Côté, R Karimi, Scopus as a curated, high-quality bibliometric data source for academic research in quantitative science studies. *Quant. Sci. Stud.* **1**, 377–386 (2020).
7. A Akbaritabar, A quantitative view of the structure of institutional scientific collaborations using the example of Berlin. *Quant. Sci. Stud.* **2**, 753–777 (2021).
8. H Hottenrott, ME Rose, C Lawson, The rise of multiple institutional affiliations in academia. *J. Assoc. for Inf. Sci. Technol.* **72**, 1039–1058 (2021).
9. H Hottenrott, C Lawson, What is behind multiple institutional affiliations in academia? *Sci. Public Policy* **49**, 382–402 (2022).
10. BC Björk, D Solomon, The publishing delay in scholarly peer-reviewed journals. *J. Informetrics* **7**, 914–923 (2013).
11. A Akbaritabar, AF Castro Torres, V Larivière, A global perspective on social stratification in science. *Humanit. Soc. Sci. Commun.* **11**, 1–10 (2024).
12. M Bell, et al., Cross-national comparison of internal migration: issues and measures. *J. Royal Stat. Soc. Ser. A (Statistics Soc.* **165**, 435–464 (2002) _eprint: https://onlinelibrary.wiley.com/doi/pdf/10.1111/1467-985X.t01-1-00247.

13. A Miranda-González, S Aref, T Theile, E Zagheni, Scholarly migration within Mexico: analyzing internal migration among researchers using Scopus longitudinal bibliometric data. *EPJ Data Sci.* **9**, 34 (2020) Number: 1 Publisher: Springer Berlin Heidelberg.
14. P Rees, et al., The Impact of Internal Migration on Population Redistribution: An International Comparison: The Impact of Internal Migration. *Population, Space Place* **23**, e2036 (2017).
15. OECD, *The Global Competition for Talent Mobility of the Highly Skilled: Mobility of the Highly Skilled.* (OECD Publishing), (2008).
16. R Core Team, *R: A Language and Environment for Statistical Computing* (R Foundation for Statistical Computing, Vienna, Austria), (2023).
17. SN Wood, *Generalized additive models: an introduction with R.* (CRC press), (2017).
18. E Pedersen, D Miller, G Simpson, N Ross, Hierarchical generalized additive models in ecology: an introduction with mgcv. *PeerJ* **7** (2019).
19. JB Lewis, DA Linzer, Estimating regression models in which the dependent variable is based on estimates. *Polit. Analysis* **13**, 345–364 (2005).
20. PC Bürkner, Bayesian item response modeling in R with brms and Stan. *J. Stat. Softw.* **100**, 1–54 (2021).
21. AS Kurz, Statistical rethinking with brms, ggplot2, and the tidyverse (2023) Accessed: 2025-01-30.
22. MG Kendall, A new measure of rank correlation. *Biometrika* **30**, 81–93 (1938).
23. A Akbaritabar, MJ Dańko, Scripts, data, and replication materials for "Global subnational estimates of migration of scientists reveal large disparities in internal and international flows" (Zenodo) (2025).
